# Role of insulin-like growth factor 1 (IGF1) in the regulation of mitochondrial bioenergetics in zebrafish oocytes: lessons from *in vivo* and *in vitro* investigations

**DOI:** 10.3389/fcell.2023.1202693

**Published:** 2023-06-30

**Authors:** Subhasri Biswas, Soumyajyoti Ghosh, Sudipta Maitra

**Affiliations:** Molecular and Cellular Endocrinology Laboratory, Department of Zoology, Visva-Bharati University, Santiniketan, India

**Keywords:** mitochondria, gonadotropin (GtH), PGC-1β/NRF-1, insulin-like growth factor 1 (IGF1), AKT/GSK3β signaling, oocyte maturation, zebrafish

## Abstract

Optimal mitochondrial functioning is indispensable for acquiring oocyte competence and meiotic maturation, whilst mitochondrial dysfunction may lead to diminished reproductive potential and impaired fertility. The role of the intra-ovarian IGF system in ovarian follicular dynamics has been implicated earlier. Although several studies have demonstrated the role of the IGF axis in facilitating mitochondrial function over a multitude of cell lines, its role in oocyte energy metabolism remains largely unexplored. Here using zebrafish, the relative importance of IGF1 in modulating oocyte mitochondrial bioenergetics has been investigated. A dramatic increase in ovarian *lhcgr* and *igf1* expression accompanied heightened ATP levels and mitochondrial polarization in full-grown (FG) oocytes resuming meiotic maturation and ovulation *in vivo*. Concomitant with elevated *igf1* expression and IGF1R phosphorylation, hCG (LH analog) stimulation of FG follicles *in vitro* prompted a sharp increase in NRF-1 and ATP levels, suggesting a positive influence of gonadotropin action on *igf1* expression vis-à-vis oocyte bioenergetics. While recombinant IGF1 administration enhanced mitochondrial function, IGF1R immunodepletion or priming with PI3K inhibitor wortmannin could abrogate NRF-1 immunoreactivity, expression of respiratory chain subunits, ΔΨ_M,_ and ATP content. Mechanistically, activation of PI3K/Akt signaling in IGF1-treated follicles corroborated well with the rapid phosphorylation of GSK3β at Ser9 (inactive) followed by PGC-1β accumulation. While selective inhibition of GSK3β promoted PGC-1β, Akt inhibition could abrogate IGF1-induced p-GSK3β (Ser9) and PGC-1β immunoreactive protein indicating Akt-mediated GSK3β inactivation and PGC-1β stabilization. The IGF1-depleted follicles showed elevated superoxide anions, subdued steroidogenic potential, and attenuated G2-M1 transition. In summary, this study highlights the importance of IGF1 signaling in oocyte bioenergetics prior to resumption of meiosis.

## Introduction

An oocyte harbors the most significant number of mitochondria than any other mammalian cell (Kim et al., 2019). Accumulating evidence from mammalian models shows that mitochondria play an inevitable role in developing functionally competent female gametes. As the fully grown follicles mature, a shift in metabolic pathways, characterized by a transition from glycolysis to oxidative phosphorylation, occurs to supply more ATP and support cytoplasmic and nuclear maturation, spindle formation, and polar body extrusion (Barbehenn et al., 1974; Babayev and Seli, 2015). During this phase, functional mitochondria increase dramatically to provide the bulk of ATP for reorganization of cytoplasmic and nuclear events including gene expression and activation of major signaling cascades (Kirillova et al., 2021). Reportedly, human primordial germ cells harbor approximately 200 copies of mtDNA, reaching 400,000 copies in a mature oocyte (Jansen, 2000). The distribution pattern of mitochondria also changes from homogenous distribution in the cytoplasm at the germinal vesicle stage to clustered aggregation near the perinuclear region in metaphase I or II oocytes (Wang et al., 2009). Earlier, a significant increase in ATP levels concomitant with mitochondrial clustering at the perinuclear area has been reported during mouse and pig oocyte maturation (Yu et al., 2010). Importantly, a maturation-dependent increase in mitochondrial membrane polarization in mouse oocytes drives ATP production and fuels cell cycle progression (Al-Zubaidi et al., 2019). A transient decrease in ATP content during meiotic maturation can disrupt spindle formation and result in meiotic errors and irregular distribution of chromosomes (Adhikari et al., 2022). Importantly, lower mitochondrial copy number accounts for poor oocyte fertilizability in humans (Reynier et al., 2001). Besides, inappropriate distribution of mitochondria has been shown to interfere with the cytoplasmic microtubule network leading to retarded or delayed oocyte maturation in mice (Wang et al., 2009). Nonetheless, the regulatory factors and the molecular mechanisms underlying oocyte energy metabolism prior to the resumption of meiotic maturation, a prerequisite for successful ovulation and spawning, still need to be better understood.

Oocyte maturation is multifactorial and involves several hypothalamic, hypophyseal, and peripheral hormones (Fallah and Habibi, 2020). Classically, gonadotropin-releasing hormone (GnRH) produced by the hypothalamus directs the release of pituitary gonadotropins, luteinizing hormone (LH) and follicle-stimulating hormone (FSH). In mammals as well as lower vertebrates, the pre-ovulatory LH surge triggers the resumption of meiosis in prophase-I arrested oocytes, followed by the remodeling of the granulosa cells, ovulation, and the release of the fertilizable eggs (Sen and Caiazza, 2013; Biswas et al., 2021). Notwithstanding, several exogenous factors like photoperiod, nutrition, temperature, humidity as well as environmental estrogens or hormones also influence female reproductive functions (Chieffi, 1984; D’Occhio et al., 2020; Wass et al., 2022). A wide range of putative intra-ovarian autocrine/paracrine factors has been implicated in regulating ovarian physiology. These include, but not limited to, insulin-like growth factors (IGFs), epidermal growth factor (EGF) family members, and transforming growth factor β (TGFβ) superfamily members (Erickson and Shimasaki, 2001; Sirotkin, 2011; Evron et al., 2015; Ipsa et al., 2019; Gershon and Dekel, 2020; Afradiasbagharani et al., 2022). More recently, the autocrine/paracrine role of ovarian GnRH and gonadotropin-inhibiting hormone (GnIH) has been demonstrated in the fish ovary (Pati and Habibi, 2000; Fallah and Habibi, 2020). IGF1 is one of the most extensively studied growth factors and has its production, reception, and site of action well established in the vertebrate ovary (Behl and Kaul, 2002). Studies on *Igf1* null mice reveal the development of immature ovaries concomitant with infertility and non-responsiveness towards exogenous gonadotropins (Kadakia et al., 2001), while *in vitro* administration of IGF1 promotes oocyte maturation, fertilization, and embryo development in bovine, canine and mice oocytes (Lorenzo et al., 1994; Toori et al., 2014; Sato et al., 2018). Importantly, IGF1R mutant female mice are sterile with diminished ovary, reduced aromatase and LH receptor levels, and increased apoptosis in the granulosa cells (Baumgarten et al., 2017). Recent years have marked the indispensable role of local autocrine/paracrine factors, in particular, Igfs, in fish ovarian physiology (Reinecke, 2010). In addition to hepatic Igfs synthesized under the influence of growth hormone (GH), extensive studies have reported the expression of the Igf system (ligands, receptors and binding proteins) in the teleost ovary downstream to gonadotropin action (Irwin and Van Der Kraak, 2012; Li et al., 2015; Biswas and Maitra, 2021). Unlike *igf3* (a gonad-specific Igf subtype in fish)*,* teleost Igf1 is evolutionarily conserved, sharing around 80% of sequence homology with mammalian counterparts at the deduced amino acid level. Studies on various piscine models have demonstrated the role of Igf1 in steroidogenesis, oocyte maturation, follicle growth and survival (Reinecke, 2010). While *igf1* is a potent regulator of oocyte maturation in red seabream (Kagawa et al., 1994), mummichog (Negatu et al., 1998), tilapia (Berishvili et al., 2006) and short-fined eel (Lokman et al., 2007), it increases the number of gap junctions and progesterone receptors in red seabream and spotted seatrout follicles for the acquisition of maturational competence (Patiño and Kagawa, 1999; Thomas et al., 2001). In the zebrafish ovary, administration of recombinant IGF1 promotes meiotic maturation potentially through increased expression and phosphorylation of epidermal growth factor receptors (Xie et al., 2016). Besides, potential synergism between maturational steroids and IGF1 to overcome prophase I arrest has been reported in zebrafish oocytes (Das et al., 2016). Despite the inevitable role of IGF1 in ovarian development and functionality, its potential involvement in gonadotropin regulation of mitochondrial bioenergetics in the oocytes undergoing meiotic G2/M1 transition is missing.

Growing evidence from non-reproductive tissues and cell lines has shown IGF1 regulation of mitochondrial function and biogenesis (Ribeiro et al., 2014; Sádaba et al., 2016; Aghanoori et al., 2019). Primarily, the mitochondrial function is governed by a set of transcription factors (NRF-1, NRF-2, ERRα, PPARs, and other nuclear factors) and nuclear coactivators belonging to the PGC-1 family (PGC-1α, PGC-1β, and PRC) (Scarpulla, 2008). While the transcription factors govern the expression of genes related to respiratory chain apparatus, mitochondrial biogenesis, and protein import, the PGC-1 family coactivators potentiate the activity of these nuclear transcription factors by enhancing their DNA-binding ability (Scarpulla, 2008). Further, AMPK and SIRT1, the two metabolic sensors, act as the gatekeepers of PGC-1α activity through phosphorylation and deacetylation (activation), respectively (Cantó and Auwerx, 2009). Importantly, several studies have documented the expression and localization of mitochondrial regulators in the vertebrate ovary. While *NRF1* and *TFAM* transcript abundance shows elevated expression during meiotic maturation in human or porcine oocytes (Antelman et al., 2008; Ghaffari Novin et al., 2015), ablation of NRF-1 gene reduced TFAM transcriptional activity, mtDNA content, and ATP production in goat granulosa cells (Zhang et al., 2017). Moreover, AMPK/SIRT1/PGC-1 network has been shown to play a central role in female reproduction, including folliculogenesis, meiotic maturation, and pregnancy maintenance (Zhou et al., 2012; Di Emidio et al., 2021; Froment et al., 2022). Nonetheless, their ability to integrate with the endocrine or autocrine/paracrine factors in the ovary remains elusive.

Zebrafish has emerged as an alternative vertebrate model for understanding the pathogenesis of human mitochondrial diseases due to high sequence similarity with human mtDNA (approximately 70%) and the same complement of nuclear mitochondrial proteins (Steele et al., 2014; Azevedo et al., 2020). Given that zebrafish ovary contains ovarian follicles at various stages of oogenesis, the relative ease of obtaining full-grown follicles throughout the year makes it an ideal model organism for exploring ovarian physiology and its regulation (Nasiadka and Clark, 2012; Hoo et al., 2016). In zebrafish and many other teleosts, resumption of meiosis requires several energy-driven processes, including germinal vesicle migration, nuclear membrane dissolution, spindle formation, and first polar body exclusion, followed by ovulation and fertilization (Lessman, 2009). Nonetheless, the dynamics of the major mitochondrial determinants and factors regulating them in full-grown (post-vitellogenic) oocytes resuming meiotic maturation and ovulation are unknown and form the basis of the present study. Results from *in vivo* investigation show that in females breeding in captivity, the presence of higher polarized mitochondria performing OXPHOS parallels the heightened expression of *lhcgr* and *igf1* prior to meiotic resumption and ovulation providing evidence favoring physiological relevance of IGF axis in oocyte mitochondrial bioenergetics. Moreover, data from *in vitro* studies reveal that while hCG (the LH analog) upregulates major mitochondrial markers in a manner sensitive to IGF1R blockade, the addition of recombinant IGF1 could augment mitochondrial function vis-à-vis cell cycle progression potentially through Akt/GSK3β signaling in zebrafish G2-arrested full-grown follicles.

## Materials and methods

### Animal collection and maintenance

Sexually mature zebrafish (*Danio rerio*) (6–12 months old) were maintained in 60 L glass aquaria, under 28°C ± 1°C and 14 h light: 10 h dark with the light signal at 06:00 (Maitra et al., 2014). Fish were fed *ad libitum* thrice daily with live blood worms and acclimatized for at least 10 days to laboratory conditions prior to their use in experiments following standardized laboratory protocols (Westerfield, 2000). All animal experiments were executed following the guidelines of the Institutional Animal Ethics Committee of Visva-Bharati University (1819/GO/Re/S/15/CPCSEA dated 31.08.2018) and approved (IAEC/III-08/2020) by the committee according to Indian law.

### Chemicals and antibodies

Recombinant human IGF1, human chorionic gonadotropin (hCG; LH analog), PI3-Kinase inhibitor wortmannin, proteinase-K, alkaline phosphatase-conjugated anti-rabbit IgG, and anti-rabbit IgG-FITC were obtained from Sigma-Aldrich, India. Dihydroethidium (DHE) and JC-1 reagent were procured from Cayman, United States. Rabbit polyclonal anti-PGC1 beta: A17258; anti-NRF1: A5547; anti-TFAM: A13552; anti-SDHA: A2594; anti-COX IV: A6564; anti-UQCRC2: A4181; anti-ATP5A1: A5884; anti-STAR: A16432; anti-LHCGR: A6266 were from ABclonal (Woburn, MA, United States). Rabbit polyclonal anti-p-PI3 Kinase p85 (Tyr458): #4228 and rabbit monoclonal anti-p-IGF-1 Receptor β (Tyr1135/1136): #3024; anti-p-Akt (Ser473): #4060; anti-p-Akt (Thr308): #9275; anti-p-AMPKα (Thr172): #2535; anti-p-GSK-3β (Ser9): #9336; anti-GSK-3β: #39315 were from Cell Signalling Technology (Danvers, MA, United States). Rabbit polyclonal anti-p-IRS1 (Tyr612): ZRB09432; anti-actin: A2066 were from Sigma-Aldrich, India. Anti-SIRT1 antibody: ARP32386 was from Aviva Systems Biology, India. TRIzol reagent was from Invitrogen, Life Technologies. Details of the antibodies used in this study and their evaluation of putative binding to the host target protein have been enlisted in Supplementary Table S1. Anti-IGFIRβ was raised in rabbit against a synthetic peptide corresponding to the C-terminus of IGF1Rβ of human origin and is identical to the corresponding region of zebrafish IGF1 receptor (Maures et al., 2002). Unless otherwise specified, hormones, inhibitors and other reagents were from Sigma-Aldrich, India.

### Kinetics of *in vivo* induction of oocyte maturation and ovulation in gravid females

Sexually mature males and females were maintained in separate aquaria at 28°C ± 1°C and 14 L:10 D cycle for at least 7 days prior to their use in the experiment (Westerfield, 2000). On the day before sampling, fish were released together in the breeding tanks at dusk (18:00) at the ratio of 1 female: 2 males (5 females with 10 males/experimental time-point) in 40 L aquaria. The maintenance and breeding of zebrafish were as described elsewhere (Nagel, 2002; Braunbeck et al., 2005; Das et al., 2016). Briefly, the bottom of the breeding tanks was replaced by a stainless-steel grid of mesh size 2 mm and placed on top of egg trays of similar dimensions for the collection of eggs after spawning. Plant imitations of green plastic wire materials were used as spawning substrates. As reported earlier (Braunbeck et al., 2005) as well as in our setup, females start releasing eggs within 30 min of morning lights on at 06:00. To cover the pre-ovulatory time frames, the females (*n* = 5/clock hour in triplicate) were autopsied at 04:00 (−2 h before light signal on), 05:00 (−1 h before light signal on), 06:00 (0 h with the light signal on), and 06:30 (+0.5 h after light signal on and just prior to ovulation). These time points provided us with ovarian follicles of immature fully grown stage with intact GV (04:00), onset of oocyte maturation (05:00), matured with complete GVBD (06:00), and peri-ovulatory follicles in the abdominal cavity (06:30) (Das et al., 2016; Liu et al., 2017). For the determination of mitochondrial parameters, ovaries were extirpated at indicated clock hours and divided into two lobes. One ovarian lobe (mid-portion) from each sampled fish was processed immediately for the quantification of ATP content and RNA isolation, whereas the other lobe was utilized for obtaining full-grown (FG) follicles for the determination of mitochondrial membrane potential.

### *In vitro* culture of zebrafish full-grown follicle-enclosed oocytes

Gravid females were anaesthetized by brief cold shock, ovaries extirpated aseptically and placed immediately in ice-cold, oxygenated zebrafish Ringer (116 mM NaCl, 2.9 mM KCl, 1.8 mM CaCl_2_, 5 mM HEPES, pH 7.2), supplemented with streptomycin (100 μg/mL) and penicillin (100 IU/mL) as described elsewhere (Rezanujjaman et al., 2020). This preparation also served as the culture medium in subsequent *in vitro* experiments. Individual ovarian follicles were separated manually using a pair of fine-tip forceps or gentle pipetting under a dissecting microscope as described earlier (Das et al., 2013). The protocol for selecting fully grown intact follicles was according to our standard laboratory protocols (Maitra et al., 2014). In brief, freshly isolated healthy FG (mean diameter ∼650 µm) follicles from independent donor females were pooled and cultured in 1 mL of Ringer’s solution in a 24-well tissue culture plate (∼50–60 follicles/well) for indicated time intervals at 25°C ± 1°C under gentle agitation (40–50 rpm). Recombinant human IGF1 and hCG were dissolved in sterile water. PI3K inhibitor wortmannin (Wort, 10 µM) was dissolved in DMSO and added to the incubation medium 1 h prior to IGF1 addition. The effectiveness of the dose selected for Wort (10 µM) was confirmed through attenuated Akt phosphorylation. All chemicals were prepared at 1000-fold stock, and oocytes in control wells received an equivalent volume of solvent only. Triton X-100 mediated delivery of anti-IGF1Rβ or anti-NRF-1 into the oocytes was performed following the method described previously (Maitra et al., 2014; Das et al., 2016). Briefly, Triton X-100 diluted (0.1%, v/v) with 10 mM PBS; pH 7.2 was sonicated for 1 min and complexed with anti-IGF1Rβ or anti-NRF-1 antibody (20 μg/mL) for 30 min each at 25°C ± 1°C and at 4°C. The resultant complex (10 µL) was layered on FG follicles in culture. The medium was replaced with a fresh Ringer after 1 h followed by hCG (10 IU) or IGF1 (10 nM) stimulation for indicated time. NaN_3_ (6.25 mM) and LiCl (15 mM) were dissolved in sterile water. Doses of hormones and inhibitors used in this study were as described earlier (Stackley et al., 2011; Das et al., 2016; Martin et al., 2018; Fallah and Habibi, 2020; Biswas and Maitra, 2021). Viability was assessed by the 0.1% trypan blue dye exclusion method. All subsequent *in vitro* experiments were carried out in triplets using pooled ovaries from separate donor females (*n* = 5).

### Determination of mitochondrial membrane potential using JC-1 staining and confocal microscopy

The mitochondrial membrane potential was determined using the fluorescent probe JC-1 (5,5′,6,6′-tetrachloro-1,1′,3,3′-tetraethyl-imidacarbocyanine iodide) staining following the manufacturer’s protocol (Cayman Chemical, United States). JC-1 depicts the mitochondrial metabolic status; while the red fluorescence due to the formation of J-aggregates indicates high membrane potential and energized mitochondria, the green fluorescence of JC-1 monomers is indicative of low membrane potential (Sivandzade et al., 2019). Briefly, follicle-enclosed oocytes harvested from independent donor females (*n* ≥ 5) were cultured in triplicate for indicated treatments and time points, followed by incubation with JC-1 dye for 30 min in the dark. Subsequently, the stained follicles were washed thrice with freshly prepared cell-based assay buffer (Cayman Chemical, United States), assembled onto groove slides, and visualized under confocal microscopy (Leica, Switzerland) through a series of optical sections. While J-aggregates were detected with filter sets designed to detect rhodamine (λ_ex_ = 516), detection of JC-1 monomers was achieved with filter sets designed to detect FITC (λ_ex_ = 488). Objectives, pinhole, filters, gain and offset, were kept constant throughout all *in vitro* experiments. The fluorescent intensity was analyzed for each oocyte by ImageJ software taking around 30 oocytes in each group from three independent experiments.

### Immunofluorescence analysis for NRF-1 localization

FG follicles (∼60 follicles/well) from indicated treatment groups (at least three replicates per treatment group) were fixed in chilled acetone overnight at 4°C, washed (x 3) with cold PBS (10 mM, pH 7.4), permeabilized with 0.5% Triton-X-100 containing PBS, and blocked in 2% BSA in PBST (PBS + 0.1% Tween 20) for overnight at 4°C. Subsequently, the follicles were washed (x 3) and incubated with NRF-1 antibody (1:50; in blocking solution) in a micro-centrifuge tube overnight at 4°C. Follicles were washed (x 3) and incubated with anti-rabbit IgG-FITC (1:250; in blocking solution) for 2 h at RT, washed and counter-stained with 1 μg/mL of 4′, 6-diamidino-2-phenylindole (DAPI; Sigma) for 1 min followed by visualization under confocal microscopy. Follicles incubated without primary antibody served as the negative control. A series of optical sections were captured for around 30 follicle-enclosed oocytes to examine NRF-1 antigen localization in individual follicular cells.

### ROS determination by dihydroethidium (DHE) oxidation assay

The determination of follicular ROS was carried out using DHE (10 μM) reactive oxygen probe followed by fluorescence microscopy as described earlier (Biswas and Maitra, 2021). Briefly, FG follicles cultured in triplicate for indicated treatments and time points were incubated with oxidation-sensitive fluorescent probe (DHE) for 30 min in the dark and observed under a fluorescence microscope (Victory FL, Dewinter Optical Inc.) using an excitation filter G (Green): 510–550 nm. The fluorescent intensity was analyzed for each oocyte by ImageJ software taking around ten fields from each replicate. The experiment was repeated thrice using FG follicles harvested from different donor females.

### Total RNA extraction, cDNA synthesis and quantitative reverse-transcriptase polymerase chain reaction (qPCR)

Total RNA was extracted from FG follicles (∼60 follicles/treatment well in triplicate) using TRIzol solution following manufacturer’s protocol. Reconstitution of RNA pellet was followed by quantification, and integrity check was performed using Multiskan Go microplate spectrophotometer (Thermo Scientific, India). 1 μg of total RNA from each treatment group pre-treated with 1 U of DNase I was reverse transcribed at 42°C for 1 h using Verso cDNA synthesis kit (#AB1453, Thermo Scientific), and qPCRs were set up on a Quant-Studio-5 Real-Time PCR System (Applied Biosystems) (Biswas et al., 2020) using gene-specific primers from earlier reports (Supplementary Table S2). The reaction mix for both target and reference genes contained cDNA template (diluted 1:10), primers (250 nM each for forward and reverse) and SYBR Green PCR Master Mix (Applied Biosystems). An initial enzyme activation step at 95°C for 10 min was followed by 45 cycles of thermal cycling with denaturation at 95°C for 30 s, annealing at 60°C for 30 s, and elongation at 72°C for 45 s. PCR conditions were optimized to produce primer amplification efficiency of 90%–110%. Briefly, each primer pair was tested using a serial dilution series of cDNA of the control group (undiluted, 1:10, 1:100, 1:1000, 1:10000). A standard curve was generated and the primer efficiency was calculated using the equation: Efficiency %E=10^−1/slope*100 . Inclusion of no template control (NTC) and a melt-curve analysis followed by agarose gel electrophoresis was executed to confirm the absence of contamination and specificity of amplification, respectively. To quantify the relative expression of each gene, the comparative threshold cycle (Ct) values for all samples were normalized for the reference gene *ef1a* as it showed stable expression over time as well as between different treatment groups (Tang et al., 2007; McCurley and Callard, 2008; Nelson and Van Der Kraak, 2010a). Relative fold-change of mRNA levels over the calibrator (quantity = 1) was calculated using the $2^{\left(-∆∆Ct\right)}$ method. The error bars display the calculated maximum (RQmax) and minimum (RQmin) expression levels representing SEM of RQ value (*n* = 3 technical replicates; *n* = 3 biological replicates) (Livak and Schmittgen, 2001).

### Isolation of DNA and quantification of mtDNA copy number

DNA was extracted from cultured follicles from indicated treatment groups at 2 h of incubation *in vitro.* The follicle-enclosed oocytes were transferred to a sterile micro-centrifuge tube, homogenized with DNA extraction buffer (pH 8) containing proteinase K (10 mg/mL), and incubated at 55°C for 2 h. After checking the integrity of DNA samples by UV absorbance (A260/280 between 1.8 and 2), mtDNA copy number was determined by qPCR using *ndi*, *coxI*, *coxII* as the mitochondrial gene target *versus* a nuclear-encoded gene *ef1α* (Supplementary Table S3). After an initial denaturation step for 10 min at 95°C, PCR amplification was executed using 100 ng DNA in a Quant Studio 5 Real-Time PCR platform and a cycling profile of 30 s at 94°C, 1 min at 60°C, 30 s at 72°C for 40 cycles followed by a melt curve analysis to check the specificity of amplification. The mtDNA content was calculated by measuring the threshold cycle ratio (ΔCt) of a mitochondrial-encoded gene (*ndi*, *coxI*, *coxII*) versus a nuclear encoded gene *ef1α*.

### Preparation of oocyte extract and immunoblot analysis

Intact follicles were harvested at appropriate time intervals, washed (x 3), and lysed in ice-cold oocyte extraction buffer as described earlier (Biswas and Maitra, 2021), supernatant separated by spinning at 17,500×g for 20 min at 4°C, and stored at 80°C until further use. Following the quantitation of protein concentration, oocyte lysates (50 µg/lane) from each treatment group were resolved through SDS-PAGE and immunoblotted following our standard laboratory protocols (Das et al., 2013; Maitra et al., 2014). The electroblotted Hybond-P PVDF membrane was blocked in 5% BSA in TBST (pH 7.6, Tris 50 mM, NaCl 150 mM, Tween-20 0.1%), incubated with primary antibody (1:1000) overnight at 4°C followed by secondary (1:2000) antibody incubation for 2 h at RT (for details see Supplementary Table S1). BCIP-NBT was used as the substrate to develop the bands, visualized, and recorded in Gel Doc apparatus (Bio-Rad). The signal intensity of target bands in each lane was measured through ImageJ software and neutralized by endogenous factor (anti-actin or respective total protein) for that lane. The normalized target signal for each sample is then divided by the normalized target signal observed in the experimental control sample to generate the fold change in target protein (*n* = 3 technical replicates; *n* = 3 biological replicates) (Pillai-Kastoori et al., 2020).

### Quantification of ATP levels

Estimation of ATP levels from indicated treatment groups (∼80 follicles/well in triplicate) was performed using ATP Colorimetric Assay Kit (Elabscience, Texas, United States) following the manufacturer’s protocol. The assay relies on the determination of phosphocreatine formed by the catalysis of ATP and creatine phosphate by creatine kinase. Absorbance was recorded at 636 nm using a microplate reader, and results (ATP content) are expressed as nmol/mg tissue.

### Kinetics of meiotic maturation *in vitro*

After harvesting and separating ovarian follicles as described in previous sections, FG follicles with mean diameter ∼650 µm and without any morphological deformity were selected under a stereo zoom microscope (Dewinter, Italy) and cultured in 24 well plates (50 oocytes/well) in the presence of hCG (10 IU) or IGF1 (10 nM) either alone or in combination with αIGF1R and NaN_3_ at 25°C ± 1°C under gentle agitation to study oocyte maturation *in vitro.* The germinal vesicle breakdown (GVBD) was monitored at 6 h through immersion in clearing solution (3% ethanol, 6% formaldehyde and 1% acetic acid) and visualizing ooplasmic clearance under an inverted microscope (Victory FL, Dewinter Optical, Inc., Italy) fitted with phase-contrast optics. These conditions were optimal in our model system and as reported earlier (Nelson and Van Der Kraak, 2010b; Zhou et al., 2016; Biswas and Maitra, 2021).

### Data analysis

All the experimental data presented are mean ± S.E.M. of at least three independent experiments. Data were analyzed by Student’s t-test (for single-factorial designs) and one-way analysis of variance (ANOVA) followed by *post hoc* multiple comparison tests (Tukey HSD and Bonferroni) (for multi-factorial designs) using GraphPad Prism8 software. *p <* 0.05 was considered statistically significant.

## Results

### Dynamics of oocyte mitochondrial parameters during *in vivo* meiotic maturation and ovulation in zebrafish

**FIGURE 1:**
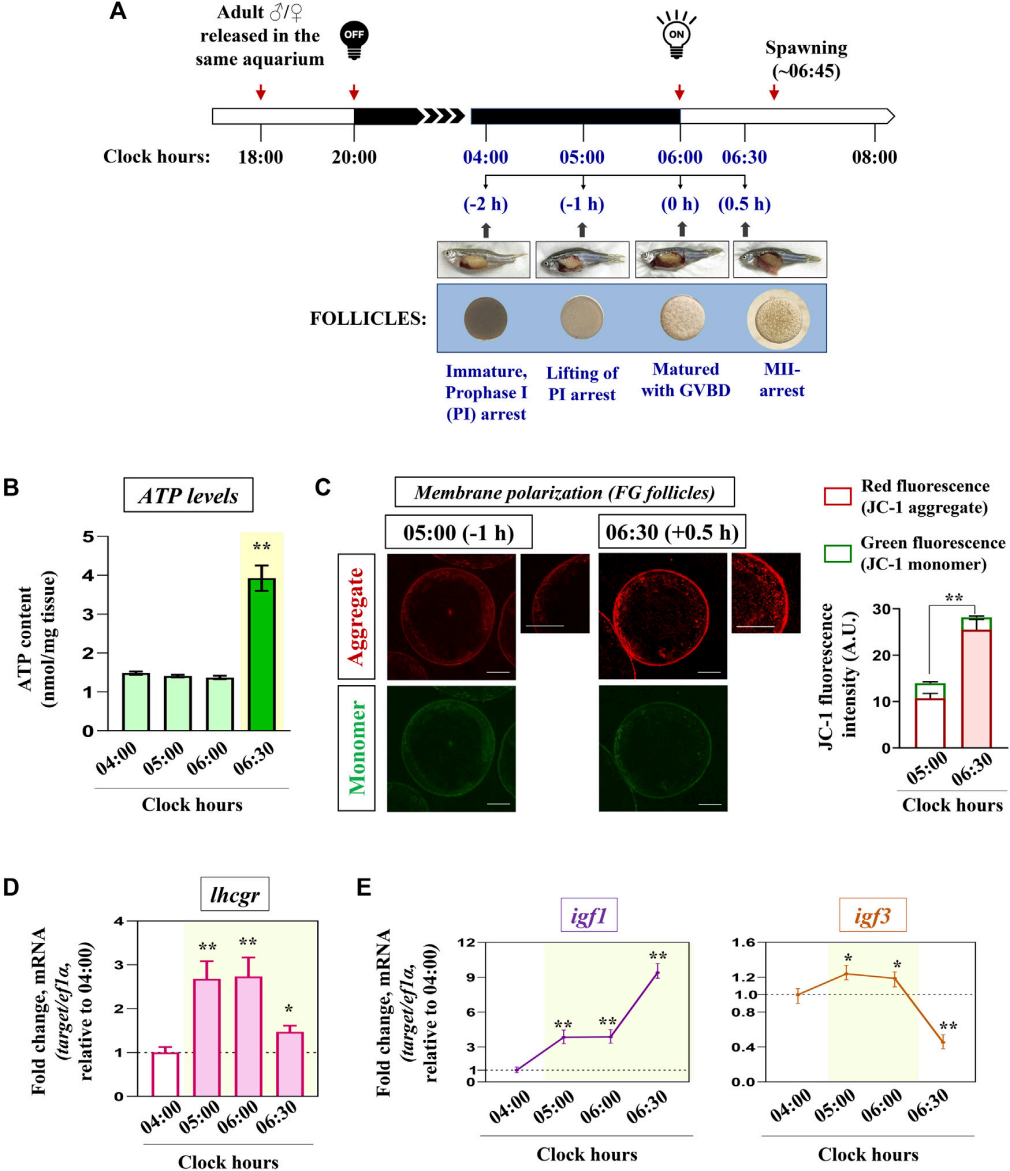
Kinetics of key markers associated with mitochondrial bioenergetics *in vivo.* Schematic representation of the time-scale for the induction of oocyte maturation followed by the ovulatory response in zebrafish *in vivo* **(A)**. Representative photomicrographs of gravid females and follicle-enclosed oocytes at indicated time points are shown (not according to scale). *n* = 3 experiments with five females for each time point. Ovaries harvested at indicated time points were subjected to ATP measurement **(B)**, and FG follicles pooled from separate donor females (n ≥ 5) were assayed for mitochondrial membrane potential through JC-1 staining **(C)**. Representative photomicrographs depicting J-aggregates (red fluorescence) and JC-1 monomers (green fluorescence) from indicated time points are shown. The relative fluorescence intensity is based on multiple (≥10) fields taking around 30 oocytes in each group (*n* = 3). Scale bar ∼200 µm. RNA samples from all four sampling hours were reverse transcribed, and the transcript abundance of *lhcgr, igf1* and *igf3* **(D, E)** were assessed through qPCR; *ef1α* served as the endogenous control. Values are mean ± S.E.M. of three independent experiments. Data are expressed relative to 04:00 **(A, B, D, E)** and 05:00 **(C)** clock hours; (*) denotes *p <* 0.05 and (**) denotes *p <* 0.01.

The dynamics of mitochondrial bioenergetics machinery during the natural course of meiotic resumption and ovulation triggered by early morning light signals was examined *in vivo*. Ovaries were harvested from gravid females (*n* = 5) at four consecutive time intervals just prior to the release of eggs (spawning): 04:00 (−2 h before light signal on), 05:00 (−1 h before light signal on), 06:00 (0 h with the light signal on), and 06:30 (+0.5 h after light signal on) (Figure 1A). These time points provided us with ovarian follicles mostly at stages of G2-arrest (04:00), initiation of maturation (05:00), matured with complete GVBD (06:00), and peri-ovulatory follicles (06:30), without using any exogenous hormonal stimulation.

Interestingly, the remarkable increment in ATP levels (Figure 1B) and formation of J-aggregates (Figure 1C) at 06:30 (i.e., just prior to the release of eggs) prompted us to examine the status of the endocrine and growth factor axis during these sampling hours. As shown in Figure 1D, *lhcgr* expression showed a sharp increment from 05:00 (−1 h) onwards when the oocytes underwent GV migration and GVBD. While *igf1* mRNA expression gradually increased from 05:00 (−1 h) onwards (∼3.8 folds), reaching the peak at 06:30 (+0.5 h) (∼9.4 folds), the transcript abundance of *igf3* displayed a marginal but significant increase (∼1.2 folds) over 04:00 (−2 h) followed by its sharp decline during the ovulatory phase (06:30) (Figure 1E). These data prompted us to hypothesize the possible involvement of gonadotropin signaling in modulating oocyte energy metabolism.

### Regulation of *igf1* expression and oocyte bioenergetics by hCG stimulation *in vitro*

**FIGURE 2:**
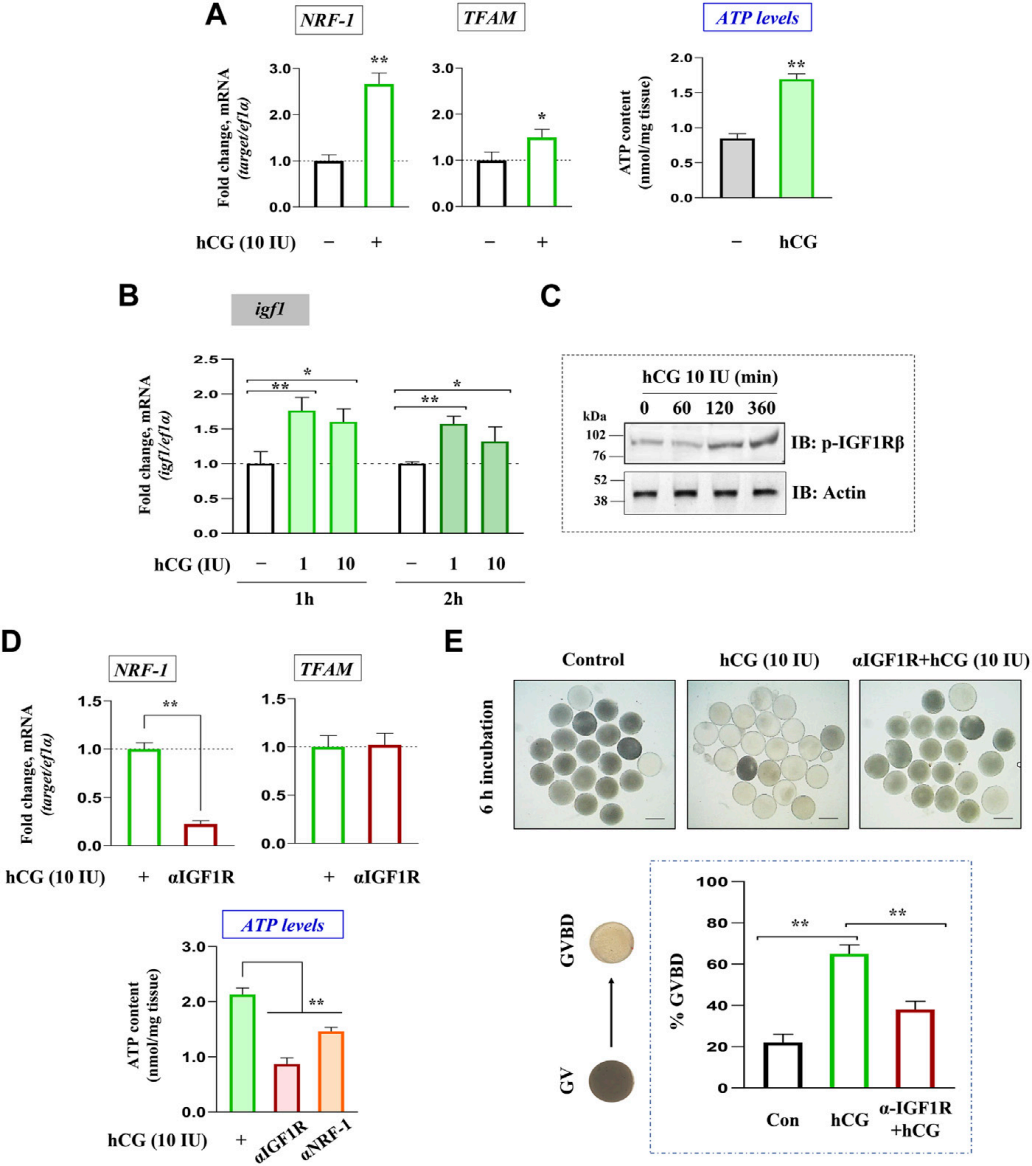
Gonadotropin-mediated regulation of *igf1* expression vis-à-vis oocyte energy metabolism. Total RNA isolated from FG follicles incubated with hCG (10 IU) for 2 h *in vitro* was reverse transcribed, followed by qPCR analysis using *NRF-1* and *TFAM* gene-specific primers (left panel, **(A)**) . Effect of hCG (10 IU) stimulation on ATP levels at 2 h of incubation *in vitro* (right panel, **(A)**). Profiling *igf1* expression in hCG-treated (1 and 10 IU) follicles at indicated time points through qPCR **(B)**. The impact of hCG stimulation on IGF1Rβ phosphorylation through immunoblot analysis at indicated time intervals **(C)**. Effect of priming (1 h) with anti-IGF1Rβ on hCG-stimulated *NRF-1* and *TFAM* expression (upper panel, **(D)**). Follicular ATP levels in IGF1Rβ or NRF-1 immunodepleted follicles compared to hCG (10 IU)-treated positive controls (lower panel, **(D)**). Besides, follicle-enclosed oocytes were primed (1 h) with anti- IGF1Rβ followed by hCG (10 IU) stimulation for 6 h, and % GVBD was determined microscopically **(E)**. Data are representative of at least three separate experiments with identical results. Results are mean ± S.E.M. (*n* = 3). Data are expressed relative to untreated **(A, B, E)** and hCG-stimulated **(D, E)** follicles; (*) denotes *p <* 0.05 and (**) denotes *p <* 0.01. Scale bar ∼500 µm.

LH regulation of *igf1* expression and mitochondrial bioenergetics was checked in FG follicles *in vitro*. Congruent with elevated ATP levels, hCG (10 IU) stimulation (2 h) could trigger a significant increase in *NRF-1* (nuclear transcription factor) and *TFAM* (mitochondrial transcription factor) expression *in vitro* (Figure 2A)*.* Interestingly, a sharp increase in *igf1* transcript abundance corroborated well with IGF1R phosphorylation in hCG-stimulated follicles at the indicated dose and duration (Figures 2B, C). Though *TFAM* expression remained largely unaltered, pre-incubation with anti-IGF1Rβ resulted in a sharp decline in *NRF-1* mRNA abundance (Figure 2D). While hCG administration promotes, priming with IGF1Rβ or NRF-1 antibodies could attenuate ATP levels and meiotic maturation even after hCG induction *in vitro* (Figures 2D, E), indicating the importance of gonadotropin regulation of oocyte bioenergetics, potentially through IGF axis, on the maturational process.

### Effect of IGF1 administration on key markers of mitochondrial bioenergetics in zebrafish FG follicles

**FIGURE 3:**
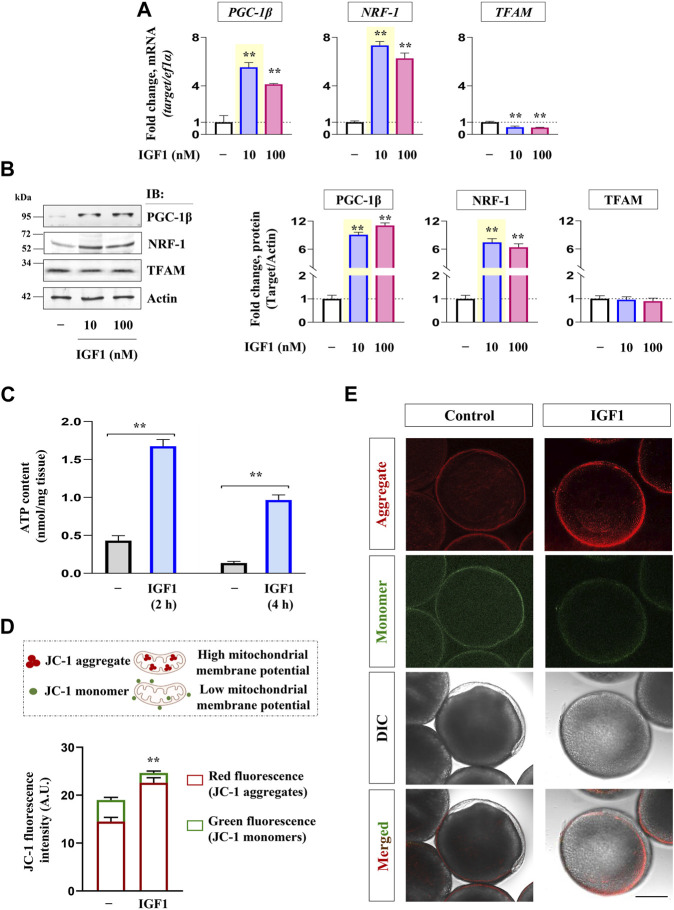
Effect of recombinant IGF1 on bioenergetics markers, ATP production and mitochondrial polarization in FG follicles *in vitro.* Profiling mRNA **(A)** and protein **(B)** expression of nuclear coactivators (*PGC-1β*) and transcription factors (*NRF-1, TFAM*) at indicated dose levels of IGF1. FG follicles pooled from separate donor females (n ≥ 5) cultured *in vitro* either without (−) or with IGF1 (10 nM) (2 h) were subjected to ATP measurement **(C)** and determination of mitochondrial membrane potential through JC-1 staining **(D, E)**. Representative photomicrographs and the relative fluorescence intensity of J-aggregates and JC-1 monomers from indicated treatment groups are shown. Fluorescence intensity is based on multiple (≥10) fields taking around 30 oocytes in each group (*n* = 3). Data are representative of at least three separate experiments with identical results and expressed relative to untreated group; (**) denotes *p <* 0.01. Scale bar ∼200 µm.

Priming with graded levels of recombinant IGF1 (10, 100 nM) could significantly increase the expression of PGC-1β and NRF-1 at both transcript and protein levels. However, TFAM expression did not undergo increment in IGF1-stimulated follicles at the indicated dose levels and time point (2 h) *in vitro* (Figures 3A, B)*.* Nevertheless, a robust increase in *PGC-1β* and *NRF-1* transcripts at the lower dose (10 nM) prompted us to select this as the lowest effective dose for subsequent experiments.

As shown in Figure 3C, IGF1 administration induced a significant increase in ATP content in FG follicles at both the time points tested over the unstimulated control. In a line of agreement, a sharp increase in red fluorescence concomitant with reduced green fluorescence was observed in IGF1-stimulated cells over the control at 2 h of incubation *in vitro* (Figures 3D, E). While the red fluorescence is characteristic of higher polarized mitochondria due to the formation of J-aggregates, less active mitochondria fluoresce green owing to the retention of JC-1 in its monomeric form. Notably, the corresponding differential interference contrast (DIC) images revealed that compared to untreated control follicles, which appeared nearly opaque (prophase I arrest), IGF1 stimulation prompted the resumption of meiotic maturation (evident from the appearance of germinal vesicle as white spot and startling clarity in the ooplasm) (Figure 3E), indicating a correlation between mitochondrial polarization (higher J aggregates), ATP synthesis and onset of meiotic maturation in IGF1-stimulated follicles *in vitro*.

### Relative importance of PI3K/Akt signaling on PGC-1β upregulation: GSK3β at the center

**FIGURE 4:**
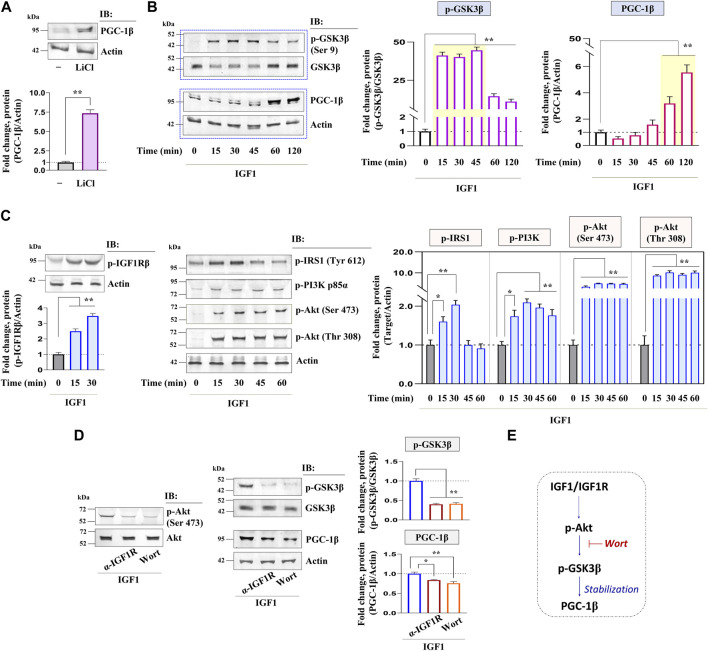
Kinetics of IGF1 activation of downstream molecular candidates: potential involvement of PI3K/Akt signaling. Effect of selective inhibition of GSK3β (using LiCl) on PGC-1β immunoreactive protein **(A)**. IGF1 stimulated follicle lysates at indicated time intervals were subjected to immunoblot analysis and probed with p-GSK3β (Ser 9), PGC-1β, p-IGF1Rβ (Tyr 1135/1136), p-IRS1 (Tyr 612), p-PI3K (Tyr 458), and p-Akt (Ser473, Thr 308) antibodies **(B, C)**. FG follicles, pre-incubated with anti-IGF1Rβ or Wort (1 h), were stimulated with IGF1 (10 nM) for 2 h and probed with p-Akt (Ser473), p-GSK3β (Ser 9), and PGC-1β antibodies **(D)**. Anti-actin and GSK3β immunoblot (total protein) served as the internal loading control. The corresponding densitometric analyses are expressed as fold change. A schematic representation depicting the impact of IGF1-mediated Akt activation on PGC-1β upregulation potentially through GSK3β inactivation **(E)**. Values are mean ± S.E.M. of three independent experiments with identical results. Data are expressed relative to untreated **(A, B, C)** and IGF1-stimulated **(D)** follicles; (*) denotes *p <* 0.05 and (**) denotes *p <* 0.01.

GSK3β is a negative regulator of the PGC-1 protein stability in somatic tissues, whether this axis is conserved in zebrafish oocytes was investigated next. As shown in Figure 4A, the selective inhibition of GSK3β by LiCl in zebrafish FG follicles could significantly upregulate PGC-1β immunoreactive protein. Correspondingly, the kinetics of GSK3β phosphorylation (Ser 9) (inhibition) revealed a sharp increase between 15 and 45 min of IGF1 stimulation, whilst the PGC-1β protein level was at its peak between 60 and 120 min of IGF1 induction (Figure 4B) indicating a close correlation between GSK3β phosphorylation (inhibition) and PGC-1β stabilization. Since GSK3β is a direct target of Akt, which phosphorylates it on Ser 9 (inactivation), we further examined the role of Akt signaling on PGC-1β upregulation in a manner sensitive to GSK3β inhibition. Immunoblot analyses revealed a rapid increase in p-IGF1Rβ (Tyr 1135/1136), p-IRS1 (Tyr 612), p-PI3K (Tyr 458), and p-Akt (Ser473, Thr 308) as early as 15 min of IGF1 (10 nM) stimulation (Figure 4C). Intriguingly, compared to IGF1-treated positive controls, pre-incubation with either anti-IGF1Rβ or wortmannin (a PI3K inhibitor) could abrogate GSK3β phosphorylation (Ser 9) as well as PGC-1β upregulation (Figure 4D) indicating that Akt signaling is essential for PGC-1β stabilization through GSK3β inactivation in zebrafish follicle-enclosed oocytes (Figure 4E).

### Impact of IGF1 deficiency on NRF-1 and mitochondrial respiratory chain subunits

**FIGURE 5:**
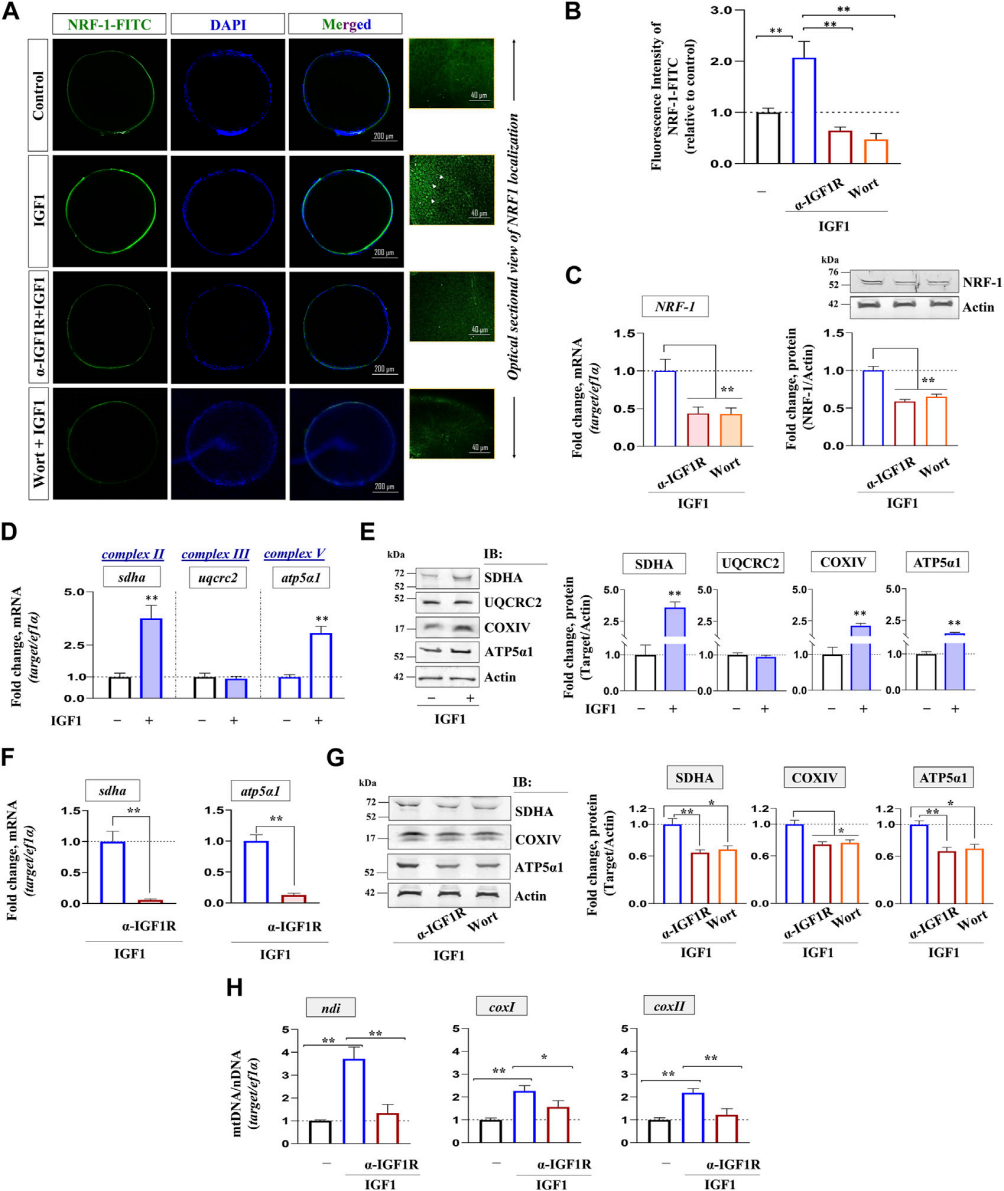
Impact of IGF1 signaling on NRF-1 and oocyte respiratory apparatus. FG follicles, either untreated or treated as indicated, were probed with NRF-1 antibody followed by anti-rabbit IgG-FITC (green), counterstained with DAPI (blue) and visualized by confocal fluorescence microscopy **(A)**. Photomicrographs are representative of at least three independent experiments with identical patterns. Arrows indicate NRF-1 immunoreactivity in individual follicular cells visualized through serial optical sections of IGF1 (10 nM)-stimulated (2 h) follicles. Corresponding fluorescence intensity from NRF-1 immunolocalized follicles from indicated treatment groups is also shown **(B)**. The relative fluorescence intensity is based on multiple (≥10) fields taking around 30 oocytes in each group (*n* = 3). Profiling gene and protein expression of NRF-1 in FG follicles primed (1 h) with anti-IGF1Rβ or Wort followed by IGF1 (10 nM) stimulation (2 h) *in vitro* through qPCR and immunoblot analysis, respectively **(C)**. qPCR analysis showing the effect of IGF1 alone and priming (1 h) with anti-IGF1Rβ or Wort on SDHA, UQCRC2, COXIV, ATP5α1 at mRNA **(D, F)** and protein level **(E, G)**. *ef1α* and anti-actin immunoblot served as the internal loading control. DNA isolated from indicated treatment groups were subjected to qPCR using gene-specific primers to examine relative abundance of mitochondrial-encoded genes (*ndi, coxI, coxII*); *ef1α* (nuclear-encoded gene) served as the endogenous control **(H)**. Values are mean ± S.E.M of three independent experiments. Data are expressed relative to untreated **(B, D, E and H)** and IGF1-stimulated **(C, F and G)** follicles; (*) denotes *p <* 0.05 and (**) denotes *p <* 0.01.

The effect of blocking IGF1 action on the expression of NRF-1, the binding partner of PGC-1β, was investigated next. Confocal fluorescence microscopy revealed a sharp increase in NRF-1 immunolocalization in IGF1-stimulated FG follicles, more specifically at the follicular layer, compared to the unstimulated group (Figures 5A, B). Serial optical sections of IGF1-stimulated follicle-enclosed oocytes showed a well-organized peripheral aggregation of NRF-1 immunofluorescence in individual follicular cells (right panel, Figure 5A). Priming with either anti-IGF1Rβ or pharmacological inhibition of PI3K/Akt signaling cascade could diminish NRF-1 expression in IGF1-treated follicles (Figures 5A, B). Corresponding immunoblot analysis revealed that compared to IGF1-treatment alone, FG follicles pre-incubated with anti-IGF1Rβ or wortmannin showed a significant downregulation in NRF-1 expression at both mRNA and protein levels (Figure 5C).

Whether IGF1 could modulate electron transport chain subunits was examined next. While IGF1 administration (10 nM) could elevate the transcript levels of nuclear-encoded (*sdha, atp5α1*) (Figure 5D) and mitochondrial-encoded (*ndi, coxI, coxII*) (Figure 5H) respiratory chain subunits, immunoblot data revealed a marked increase in SDHA, COXIV, and ATP5α1, but not UQCRC2, in the treated group over the untreated control (Figure 5E). Notably, a sharp decline in nuclear- and mitochondrial-encoded respiratory chain subunits due to the blockade of the IGF1 axis (Figures 5F–H) might indicate reduced OXPHOS activity and corroborated well with reduced NRF-1 immunolocalization under similar conditions (Figures 5A–C).

### Effect of blocking IGF1R or NRF-1 action on mitochondrial polarization and ATP levels

**FIGURE 6:**
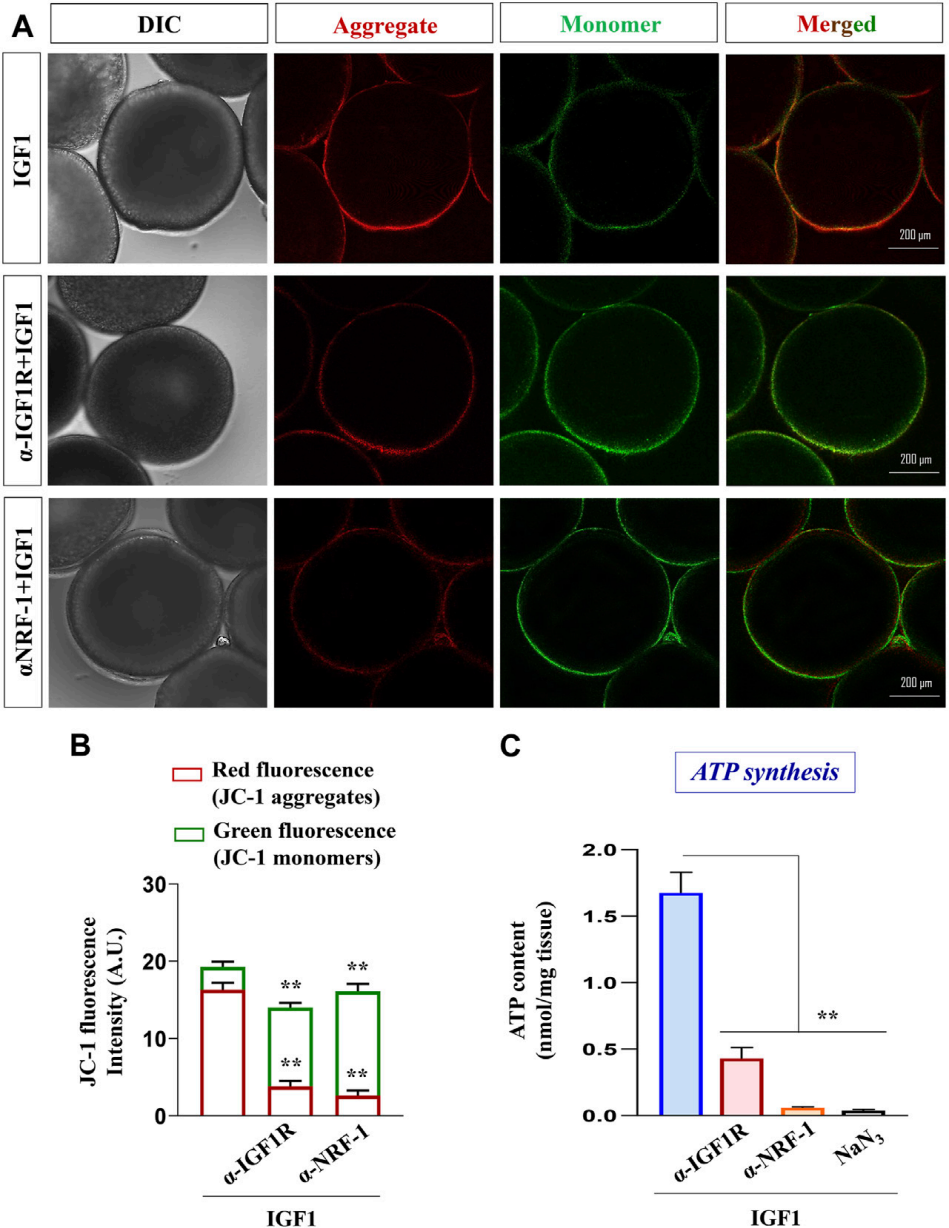
Effect of blocking IGF1 action on mitochondrial polarization and ATP synthesis. Assessment of mitochondrial membrane potential in IGF1 (10 nM)-stimulated (2 h) follicles pre-incubated with anti-IGF1Rβ and anti-NRF-1 antibodies **(A)**. The data on the lower panel is based on the relative fluorescence intensity of J-aggregates and JC-1 monomers in multiple (≥30) follicles from indicated treatment groups **(B)**. Besides, ATP levels were quantified in IGF1-stimulated (2 h) follicles, either primed (1 h) or not with anti-IGF1Rβ and anti-NRF-1 antiserum. Follicles treated with NaN_3_ served as negative control **(C)**. Data are representative of at least three separate experiments with identical results. Results are mean ± S.E.M. (n = 3); (**) denotes *p <* 0.01.

Next, the specificity of IGF1 action and the importance of NRF-1 on mitochondrial metabolic status (membrane polarization) were examined. As shown in Figures 6A, B, priming with either anti-IGF1Rβ or anti-NRF-1 prior to IGF1-stimulation resulted in the loss of red fluorescence of J-aggregates and elevated green fluorescence (JC-1 monomers), indicating low polarized organelles. Congruently, similar to the effect of NaN_3_ (an inhibitor of the respiratory chain), pre-incubation with IGF1R or NRF-1 antibodies could lower ATP levels compared to IGF1 alone (Figure 6C), indicating the importance of IGF1-mediated signaling cascade in oocyte energy metabolism.

### Altered redox homeostasis, steroidogenic potential, and maturational response in IGF1-deficient oocytes

**FIGURE 7:**
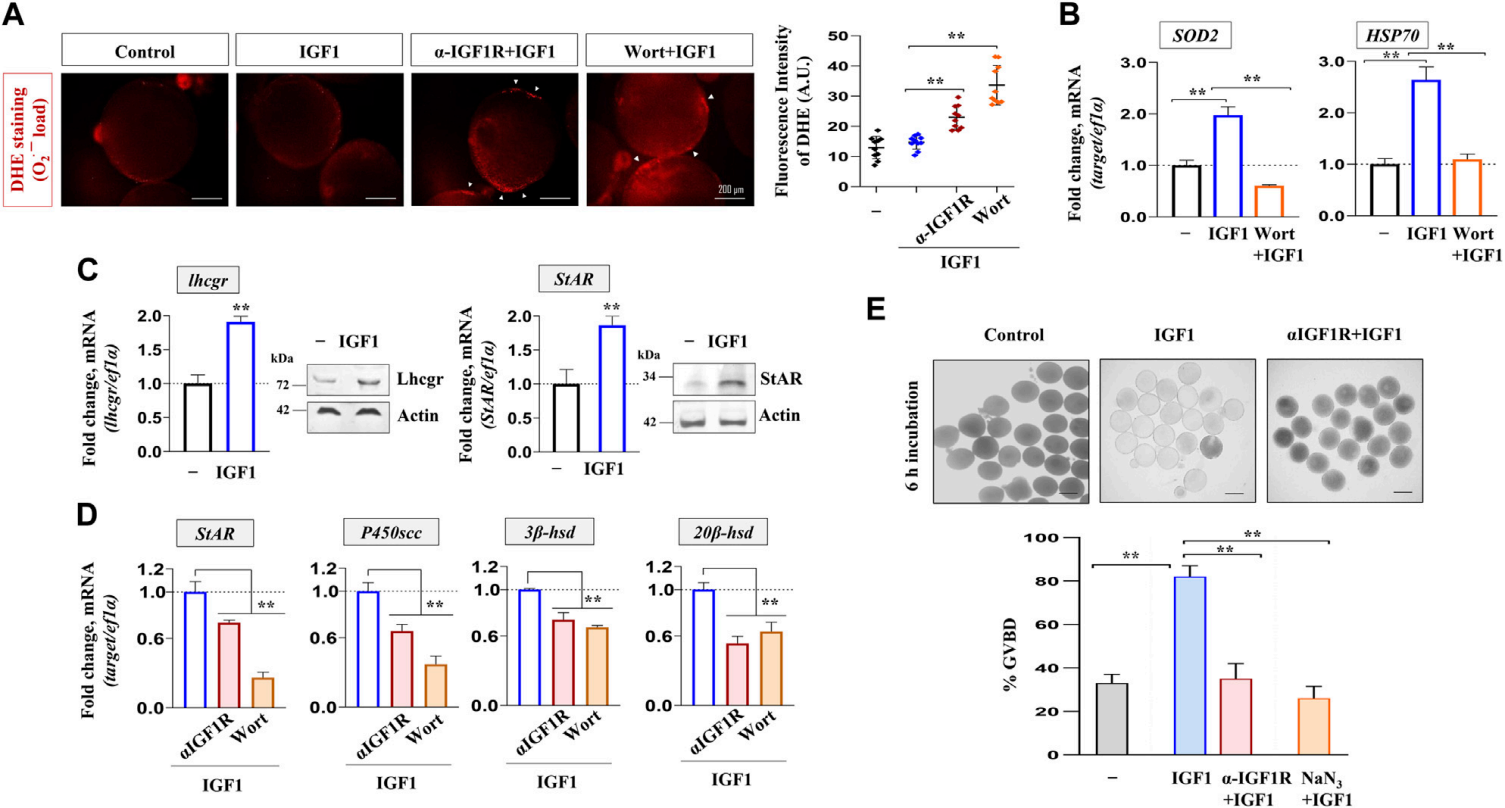
Altered redox balance, steroidogenic potential, and cell cycle progression in IGF1-deficient FG follicles. ROS measurement through DHE staining (30 min) from follicle-enclosed oocytes primed (1 h) with anti-IGF1Rβ or Wort followed by IGF1 (10 nM)-stimulation for 2 h *in vitro* **(A)**. Representative photomicrographs from fluorescence microscopy and the fluorescence intensity from DHE-stained follicles from various treatment groups are shown. Arrows indicate ROS detection at the follicular layer. Effect of priming (1 h) with Wort on *SOD2* and *HSP70* expression in IGF1 (10 nM)-treated follicles *in vitro* **(B)** *; ef1α* served as endogenous control. Intact follicles were incubated in the absence or presence of IGF1 (10 nM) followed by the determination of transcript and protein levels of Lhcgr and StAR through qPCR and immunoblot analysis **(C)**, respectively. Effect of priming (1 h) with anti-IGF1Rβ or Wort on *StAR, P450scc, 3β-hsd* and *20β-hsd* expression in IGF1 (10 nM)-treated follicles *in vitro* **(D)**. *ef1α* and anti-actin immunoblots served as the internal loading control. Besides, follicle-enclosed oocytes, primed (1 h) with either anti- IGF1Rβ or NaN_3_ (6.25 mM), were treated with IGF1 (10 nM) for 6 h and % GVBD was determined microscopically **(E)**. Scale bar ∼500 µm. Data are mean ± S.E.M of three independent experiments. Data are expressed relative to untreated **(A, B, C and E)** and IGF1-stimulated **(D)** follicles; (**) denotes *p <* 0.01.

The relevance of IGF1 action or the attenuation of it on follicular redox homeostasis, steroidogenic potential and the meiotic maturational response was investigated next. As shown in Figure 7A, the pharmacological inhibition of PI3K/Akt signaling heightened follicular levels of ROS synthesis as evident through DHE staining followed by fluorescence microscopy. Congruently, the transcript abundance of *SOD2* (a mitochondria-specific antioxidant enzyme) and *HSP70* (a ubiquitous molecular chaperone) underwent a sharp decline in wortmannin-treated follicles (Figure 7B). Besides, while IGF1 alone could elicit Lhcgr and StAR expression at mRNA and protein levels (Figure 7C), priming with anti-IGF1Rβ or Wort prevented IGF1-induced *StAR, P450scc, 3β*-*hsd,* and *20β*-*hsd* expression (Figure 7D). Intriguingly, similar to the attenuated meiotic maturational response upon NaN_3_ treatment, IGF1R immunodepleted follicles failed to undergo meiotic resumption upon IGF1 stimulation *in vitro* (Figure 7E). Thus, IGF1 regulation of NRF-1/PGC1β expression and mitochondrial bioenergetics strongly indicate the physiological relevance of functional and active mitochondria in maintaining redox homeostasis, steroid biosynthesis, and oocyte maturation in zebrafish FG follicles.

## Discussion

The development of functionally competent female gametes involves several energy-driven metabolic and synthetic processes (KofiArhin et al., 2018). Studies in mammalian models have provided ample evidence to demonstrate that a burst of energy release is a prerequisite for successful ovulation and the release of female gametes competent to undergo fertilization (Chiaratti et al., 2018). Resumption of meiotic maturation in the majority of fish species studied so far requires several energy-driven processes, including *de novo* synthesis of cyclin B from stored mRNA derived from maternal sources, phosphorylation of cdc2 at Thr161 and histone H1 kinase activation, nuclear membrane dissolution, spindle formation, and first polar body exclusion (Nagahama and Yamashita, 2008). However, there is no information on the oocyte energy metabolism in fish models before meiotic resumption and ovulation. This led us to check the status of oocyte bioenergetics during the natural course of *in vivo* meiosis resumption in female zebrafish. Ovaries have been harvested at four consecutive time points which provided us with follicles at stages of G2-arrest (04:00), maturational phase (05:00), matured with complete GVBD (06:00), and peri-ovulatory follicles (06:30) in the ovarian lumen, without using any exogenous hormonal stimulation. Our results demonstrate elicited ATP levels at 06:30, i.e., when most oocytes have completed the G2-M1 transition and are arrested at the MII stage. Confocal microscopy of FG follicles stained with the mitochondrial membrane potential-sensitive dye JC-1 shows a steep rise in the red fluorescence predominantly in the surrounding follicular layer indicating higher polarized active mitochondria performing OXPHOS prior to ovulation. This is the first report demonstrating the mitochondrial metabolic status vis-à-vis ATP synthesis in zebrafish ovaries undergoing rapid changes in the follicular microenvironment during the progression to peri-ovulatory phase. Previously, de Paula and co-workers (De Paula et al., 2013) demonstrated a decreased mitochondrial polarization in the later stages of zebrafish oocytes compared to that in follicle cells. This provided the notion of two distinct subpopulations of oocyte mitochondria: the follicle’s energy-transducing mitochondria and the oocyte’s bioenergetically suppressed mitochondria. Thus, it seems highly probable that the follicular cell layer remodels its energy metabolism in favor of the gamete and loads it with adequate ATP before it loses contact with the follicular cells during ovulation. A more recent study demonstrates that maturing and late-stage oocytes possess fully functional complex I and OXPHOS activity compared to early (primordial stage) oocytes in humans and *Xenopus* (Rodríguez-Nuevo et al., 2022). Nonetheless, the signaling molecules awakening the bioenergetics machinery of diplotene-arrested oocytes remain elusive.

Data from the present study demonstrate that *lhcgr* expression heightens prior to meiosis resumption. Congruently, unlike *igf3,* the expression of *igf1* undergoes a dramatic upsurge reaching its peak just before ovulation. This precedent expression of *lhcgr* followed by the robust increase in *igf1* transcripts, hints towards the probable participation of maturational gonadotropin in IGF1 synthesis vis-à-vis mitochondrial bioenergetics. G2-arrested (immature) follicles were cultured *in vitro* and stimulated with hCG (LH mimicking hormone) to test this hypothesis. Earlier, hCG at 10 IU/mL has been shown to effectively stimulate GVBD in zebrafish full-grown follicles (Fallah and Habibi, 2020; Biswas and Maitra, 2021), prompting us to select this particular dose. While hCG heightens *igf1* expression and IGF1R phosphorylation (activation) concomitant with increased *NRF-1, TFAM*, and ATP levels, intriguingly, priming with anti-IGF1Rβ antibody could abolish the positive influence of hCG stimulation on *NRF-1* and ATP levels. Although hCG-mediated transcriptional activation of IGF ligands has been reported earlier (Li et al., 2015; Biswas and Maitra, 2021), the role of gonadotropin in ovarian mitochondrial dynamics is being reported for the first time in zebrafish oocytes. Moreover, the physiological relevance of functional and active mitochondria in hCG stimulated cells to resume meiotic maturation in a manner sensitive to IGF1R blockade has also been demonstrated. While these results suggest that IGF1 mediates LH action on oocyte energy metabolism, it further prompted us to delineate its specificity and mode of action in zebrafish follicle-enclosed oocytes.

Interestingly, IGF1 administration upregulates the expression of PGC-1β (nuclear coactivator) and NRF-1 (nuclear transcription factor) but fails to induce TFAM (mt transcription factor) levels indicating that hCG-mediated TFAM expression is independent of IGF1 action. Although *PGC-1α* transcript was detectable in the ovary, its expression level was too low (Ct ≥ 40) for further consideration (data not shown). Reportedly, the incurred mutations in the presumptive NRF-1 binding domain of PGC-1α protein in fish lineages are possibly compensated with other PGC-1 paralogs, e.g., PGC-1β in regulating mitochondrial bioenergetics (Lemoine et al., 2010; Bremer et al., 2016). Besides, through luciferase reporter assays, PGC-1β has been shown to interact with and coactivate NRF-1 directly (Lin et al., 2002), indicating the PGC-1β isoform may be at the helm of events and binds NRF-1 to regulate mitochondrial function in zebrafish oocytes. However, selective knockdown of PGC1 isoforms vis-à-vis mitochondrial biogenesis may provide further insights. In the line of agreement, data of the present study show that concomitant with the sharp increase in ATP content, JC-1 staining followed by confocal microscopy reveals improved mitochondrial membrane potential (ΔΨ_M_) characterized by reduced green fluorescence and heightened red fluorescence (J-aggregates) in IGF1-administered FG follicles. While the presence of mitochondria with almost similar green and red fluorescence intensity has been reported in early and vitellogenic stage zebrafish oocytes (Zhang et al., 2008; Zampolla et al., 2009), our data show consistently higher J-aggregates over JC-1 monomers in FG follicles. Importantly, the red fluorescent J-aggregates are formed when the JC-1 dye enters and accumulates in the energized and negatively charged mitochondria (Sivandzade et al., 2019). Based on above, it seems highly probable that IGF1-induced mitochondrial polarization is essential for sustained ATP production at the follicular cell layer to suffice oocyte needs during maturation and ovulation.

The signaling events regulating PGC-1 family proteins downstream of IGF1 action have been examined next. Present data demonstrate that IGF1 promotes a robust increase in IGF1Rβ/IRS1/PI3K/Akt phosphorylation as early as 15 min of stimulation. Reportedly, GSK3β is a direct target of Akt, which phosphorylates the former on Ser9 leading to its inactivation and loss of kinase activity (Theeuwes et al., 2017). Besides, GSK3β is a negative regulator of PGC-1 protein stability (Martin et al., 2018). These reports conform with the results of the present study showing that GSK3β is rapidly phosphorylated with its peak levels between 15 and 45 min of IGF1 stimulation, followed by a sharp increment in PGC-1β accumulation between 60 and 120 min of IGF1 stimulation. Furthermore, while selective inhibition of GSK3β elicits PGC-1β expression over untreated control, the pharmacological inhibition of PI3K/Akt signaling abrogates GSK3β phosphorylation on Ser9 as well as PGC-1β abundance. Earlier, GSK3β inhibition using LiCl has been shown to induce the PGC-1 signaling network and potentiate mitochondrial metabolism in vascular smooth muscle, endothelial and neuronal cells (Struewing et al., 2007; Wang et al., 2013; Martin et al., 2018). Nonetheless, present study provides the first evidence demonstrating that Akt signaling is essential for stabilizing the PGC-1β family coactivator through GSK3β phosphorylation (inactivation) in zebrafish follicle-enclosed oocytes. We also have data showing the plausible involvement of AMPK and SIRT1, the two metabolic sensors that regulate PGC-1 activity through phosphorylation and deacetylation, respectively (Cantó and Auwerx, 2009). Although SIRT1 levels remain largely unaffected, present data reveal that IGF1 induces a significant increase in AMPK phosphorylation at 60 and 120 min of incubation *in vitro* in a manner sensitive to Akt inhibition (Supplementary Figure S1)*.* Since PI3K/Akt signaling has been implicated in the breakdown of cAMP into AMP in zebrafish oocytes (Das et al., 2013), the high [AMP]:[ATP] ratio might activate AMPK (Fogarty et al., 2010) in the ovarian microenvironment. However, the elaborative role of AMPK and other sirtuins in regulating the activation of the PGC-1β protein requires further exploration.

The impact of PI3K/Akt signaling on NRF-1, the nuclear transcription factor and the binding partner of PGC-1β, has been assessed. In conformity with J-aggregates’ localization, the well-organized NRF-1 immunolocalization in the polygonal shaped granulosa cells in IGF1-stimulated follicles (shown by confocal microscopy) signifies a positive concert between nuclear and mitochondrial compartment. To bridge the gap between increased PGC-1β/NRF-1 expression and follicular ATP levels, we checked whether IGF1 administration influences the oocyte respiratory apparatus. Intriguingly, results of the present study show that IGF1 upregulates NRF-1-regulated electron transport chain subunits (SDHA, COXIV, ATP5α1), whilst blocking IGF1 action through IGF1R immunodepletion or Akt inhibition results in diminished expression of the respiratory chain subunits. The next query has been whether this phenomenon involves mitochondrial biogenesis or a change in mitochondrial content. Surprisingly as mentioned earlier, although the expression status of TFAM (the promoter of mtDNA transcription and replication) did not change, the mitochondrial-encoded genes (*ndi, coxI, coxII*) underwent a sharp increase in IGF1-administered cells and showed sensitivity to IGF1R blockade. Previously, several experimental systems have documented the limited predictive value of TFAM in mitochondrial biogenesis wherein TFAM expression fails to parallel mtDNA copy number as well as the abundance of mtDNA-encoded genes (Kozhukhar and Alexeyev, 2019). Besides, the mitochondrial transcription factor B paralogs (mtTFB1 and mtTFB2) are known to activate the transcription of human mtDNA (Falkenberg et al., 2002). However, at this point, it is hard to conclude the role of mtTFBs in zebrafish oocyte mitochondrial metabolism warranting the need for future studies.

The most remarkable finding of the present study is the biological significance of IGF1 signaling in oocyte biology. Our results reveal three significant findings: 1. IGF1 normalizes mitochondrial ROS production and prevents oxidative damage of oocytes by elevating *SOD2* and *HSP70* expression in a PI3K/Akt-mediated pathway; 2. Improved mitochondrial activity is correlated to enhanced steroidogenic potential in IGF1-stimulated FG follicles; 3. Blocking mitochondrial function directly (using a selective, ETC., blocker) or through the blockade of IGF1/IGF1R reduces mitochondrial polarization and ATP synthesis, thereby attenuating meiosis resumption in G2-arrested FG follicles. These data indicate the functional relevance of functional and active mitochondria in IGF1 regulation of redox homeostasis, steroidogenic potential and maturational response *per se* in zebrafish maturing oocytes.

**FIGURE 8:**
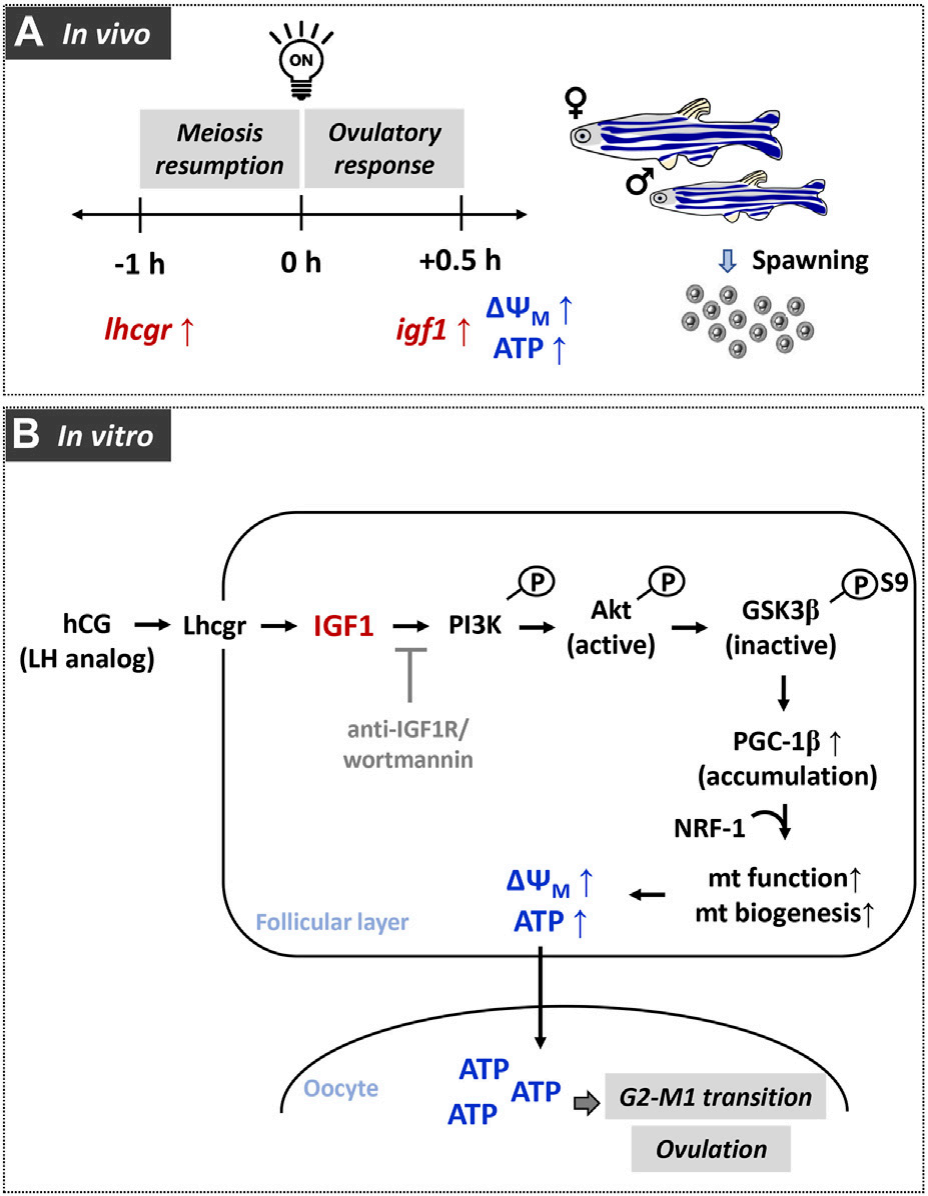
A graphical representation of the role of gonadotropin-mediated IGF1 signaling in modulating oocyte energy metabolism and converting immature oocytes to fertilizable eggs in zebrafish. In females undergoing daily spawning cycle **(A)**, elevated expression of gonadotropin receptor (*lhcgr*) and insulin-like growth factor 1 (*igf1*) correlate well with higher polarized mitochondria and ATP content in FG follicles. Data from *in vitro* experiments **(B)** reveals participation of Akt/GSK3β/PGC-1β signaling during IGF1 induction of mitochondrial polarization as well as OXPHOS activity.

**FIGURE 9:**
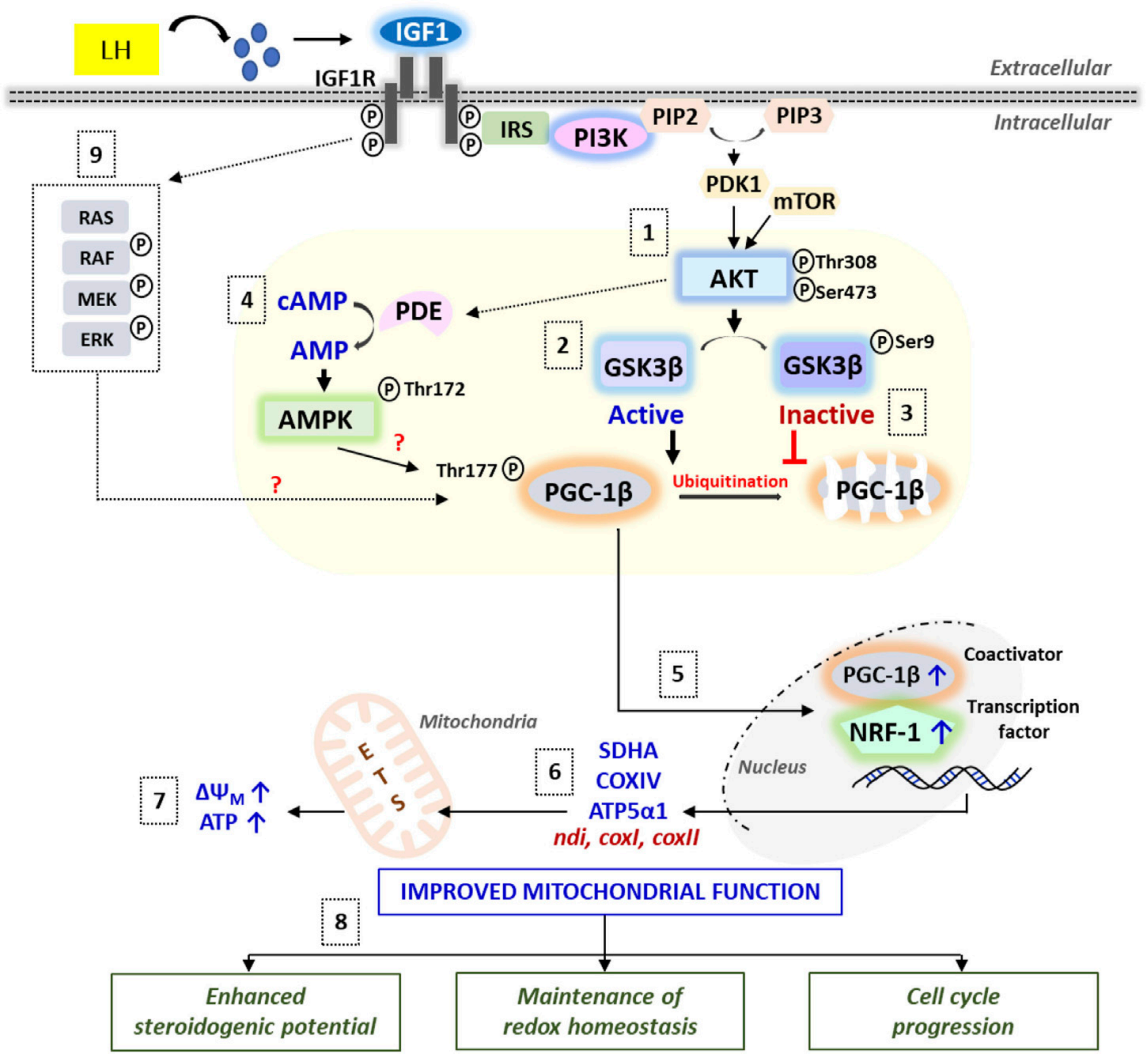
A schematic illustration of the IGF1-mediated signaling cascades regulating mitochondrial bioenergetics vis-à-vis follicular events in zebrafish follicle-enclosed oocytes. Synthesized under the influence of LH action, IGF1 binding to its cognate receptor IGF1R leads to rapid activation of PI3K/Akt signaling (1), which in turn phosphorylates GSK3β resulting in its inactivation (2) and prevention of proteasomal degradation of PGC-1β protein (3). Besides, Akt-mediated rapid breakdown of cAMP into AMP (potentially through PDE activation) might stimulate AMPK, which strongly influences PGC-1β phosphorylation and activation (4). PGC-1β partners with NRF-1 and the resultant complex translocates into the nucleus (5), leading to the transcriptional activation of electron transport chain subunits (6) and improved ATP production (7). Cumulatively, IGF1-mediated improvement in mitochondrial function converges into enhanced steroidogenic potential, balanced redox state, and meiotic maturation in zebrafish maturing oocytes (8). Nonetheless, the role of IGF1-mediated activation of the MEK/ERK pathway on oocyte bioenergetics requires more in-depth investigations (9).

In conclusion, the relative importance of IGF1 signaling in regulating mitochondrial bioenergetics has been demonstrated in zebrafish follicle-enclosed oocytes both *in vivo* and *in vitro* (Figure 8). Data of the present study strongly supports the active operation of the mitochondrial bioenergetic machinery during the natural course of oocyte maturation and ovulation in this species*.* Most importantly, the paralleled *igf1* expression reaching the peak just prior to ovulation indicates plausible participation of this ovarian growth factor in oocyte bioenergetics. Results from *in vitro* studies further revealed that recombinant IGF1 upregulates the major mitochondrial markers, mitochondrial polarization, and ATP synthesis in a manner sensitive to PI3K/Akt signaling cascade. Improved steroid biosynthesis and acquisition of G2-M1 transition attest to the functional relevance of energized and active mitochondria (Figure 9)*.* This study provides a better understanding of the metabolic networks regulating oocyte bioenergetics during maturation and ovulation utilizing zebrafish, an emerging model with high utility in mitochondrial toxicity and bioenergetics research. Moreover, this study may help delineate the altered energy metabolism in reproductive disease models, e.g., PCOS, ovarian ageing, and metabolic stress.

## Figures and Tables

**Table:** 

**20β-hsd**	20-beta-hydroxysteroid dehydrogenase
**3β-hsd**	3-beta-hydroxysteroid dehydrogenase
**Akt**	Ak strain transforming
**AMP**	Adenosine monophosphate
**AMPK**	5′adenosine monophosphate-activated protein kinase
**ANOVA**	Analysis of variance
**ATP**	Adenosine triphosphate
**ATP5α1**	ATP synthase F1 subunit alpha, mitochondrial
**BCIP-NBT**	5-bromo-4-chloro-3-indolyl phosphate (BCIP)/nitro blue tetrazolium (NBT)
**BSA**	Bovine serum albumin
**cAMP**	Cyclic adenosine monophosphate
**COXI**	Cytochrome c oxidase subunit I
**COXII**	Cytochrome c oxidase subunit II
**COXIV**	Cytochrome c oxidase subunit IV
**DAPI**	4′,6-diamidino-2-phenylindole
**DHE**	Dihydroethidium
**DMSO**	Dimethyl sulfoxide
**ERRα**	Estrogen-related receptor α
**FG**	Full-grown
**FITC**	Fluorescein isothiocyanate
**G2-M1**	Growth phase 2/Meiosis I
**GSK3β**	Glycogen synthase kinase 3-beta
**GVBD**	Germinal vesicle breakdown
**hCG**	Human chorionic gonadotropin
**HSP70**	70 kDa heat shock protein
**IGF1**	Insulin-like growth factor 1
**IGF1R**	Insulin-like growth factor 1 receptor
**IRS1**	Insulin receptor substrate 1
**JC-1**	5,5′,6,6′-tetrachloro-1,1′,3,3′-tetraethyl-imidacarbocyanine iodide
**LH**	Luteinizing hormone
**Lhcgr**	Luteinizing hormone/choriogonadotropin receptor
**LiCl**	Lithium chloride
**mtDNA**	Mitochondrial DNA
**NaN3**	Sodium azide
**NRF-1**	Nuclear respiratory factor 1
**NTC**	No template control
**P450scc**	Cholesterol side-chain cleavage enzyme, cytochrome P450 monooxygenase
**PGC-1α**	Peroxisome proliferator-activated receptor-gamma coactivator (PGC)-1 alpha
**PGC-1β**	Peroxisome proliferator-activated receptor-gamma coactivator (PGC)-1 beta
**PI3K**	Phosphoinositide 3-kinases
**PPARs**	Peroxisome proliferator-activated receptors
**PRC**	PGC-1 related protein
**PVDF**	Polyvinylidene fluoride
**ROS**	Reactive oxygen species
**RT**	Room temperature
**qPCR**	Real-time quantitative reverse transcription PCR
**SE.M.**	Standard error of mean
**SDHA**	Succinate dehydrogenase complex flavoprotein subunit A
**SDS-PAGE**	Sodium dodecyl-sulfate polyacrylamide gel electrophoresis
**SIRT1**	Sirtuin (silent mating type information regulation 2 homolog) 1
**SOD2**	Superoxide dismutase 2, mitochondrial
**StAR**	Steroidogenic acute regulatory protein
**TBST**	Tris buffered saline with tween
**TFAM**	Transcription factor A, mitochondrial
**UQCRC2**	Cytochrome b-c1 complex subunit 2, mitochondrial
**UV**	Ultraviolet
**Wor**t	Wortmannin
**ΔΨM**	Mitochondrial membrane potentia

## Data Availability

The original contributions presented in the study are included in the article/Supplementary Material, further inquiries can be directed to the corresponding author.
