# Supplementary material for: Role of insulin-like growth factor 1 (IGF1) in the regulation of mitochondrial bioenergetics in zebrafish oocytes: lessons from in vivo and in vitro investigations
Source: Front Cell Dev Biol. 2023 Jun 30;11:1202693. doi: 10.3389/fcell.2023.1202693 (PMC10347385; doi:10.3389/fcell.2023.1202693)
Supplement: Supplementary file 1 [file Table1.docx]

***Supplementary Material***

**Role of insulin-like growth factor 1 (IGF1) in the regulation of mitochondrial bioenergetics in zebrafish oocytes: lessons from *in vivo* and *in vitro* investigations**

***Journal: Frontiers in Cell and Developmental Biology***

***Section: Molecular and Cellular Reproduction***

Subhasri Biswas, Soumyajyoti Ghosh, Sudipta Maitra^*^

Molecular and Cellular Endocrinology Laboratory, Department of Zoology, Visva-Bharati University, Santiniketan, West Bengal, India

^*^Corresponding author

Tel.: +91 9874405555, +91 8116978904

Email: s.maitra@visva-bharati.ac.in, smaitra3@gmail.com

**Supporting Information**

**Pages: 7**

**Figure: 1**

**Tables: 3**


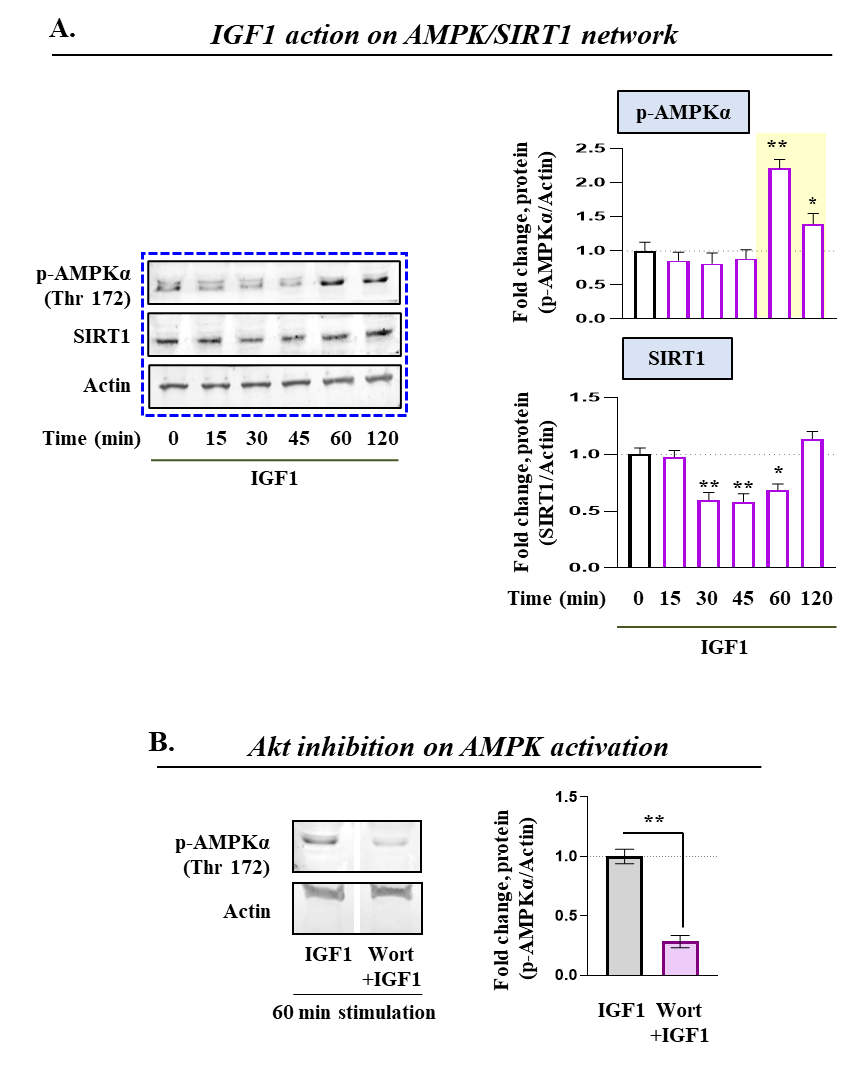


**Fig. S1. IGF1 regulation of AMPK/SIRT1 network.** FG follicles were treated with IGF1 (10 nM), and follicle lysates from indicated time intervals were subjected to immunoblot analysis and probed with p-AMPKα (Thr 172) and SIRT1 antibodies (A). Effect of priming (1 h) with Wort (10 µM) on IGF1-stimulated p-AMPKα (Thr 172) (B). Anti-actin immunoblot (total protein) served as the internal loading control. The corresponding densitometric analyses are expressed as fold change. Values are mean ± S.E.M. of three independent experiments with identical results. Data are expressed relative to untreated (A) and IGF1-stimulated (B) follicles; (*) denotes *P<* 0.05 and (**) denotes *P<* 0.01*.*

**Table S1. Evaluation of % sequence identity between immunogenic peptides and respective target proteins (*D. rerio*) through NCBI protein database and ClustalW platform.**

| Antibody | Format | Immunogen | % Sequence identity with zebrafish | Deduced molecular weight (kDa) |
| --- | --- | --- | --- | --- |
| 1. PGC-1β | Polyclonal, immunopurified, raised in rabbit | A synthetic peptide corresponding to a sequence within amino acids 850-950 of human PGC1 beta | 40.91 %  XP_009289336.1 | ~ 94.7 kDa  (https://www.uniprot.org/uniprot/A0A0R4IGS9) |
| 2. NRF-1 | Polyclonal, immunopurified, raised in rabbit | Recombinant fusion protein containing a sequence corresponding to amino acids 240-449 of human NRF-1 | 92.54 %  NP_001315469.1 | ~ 54.7 kDa  (https://www.uniprot.org/uniprot/Q90X44) |
| 3. TFAM | Polyclonal, immunopurified, raised in rabbit | Recombinant fusion protein containing a sequence corresponding to amino acids 1-220 of human TFAM | 41.06 %  NP_001070857.1 | ~ 31.4 kDa  (https://www.uniprot.org/uniprot/F1QF85) |
| 4. SDHA | Polyclonal, immunopurified, raised in rabbit | Recombinant fusion protein containing a sequence corresponding to amino acids 385-664 of human SDHA | 78.21 %  NP_957204.1 | ~ 72.6 kDa  (https://www.uniprot.org/uniprot/Q6TNQ9) |
| 5. COX IV | Polyclonal, immunopurified, raised in rabbit | Recombinant fusion protein containing a sequence corresponding to amino acids 23-169 of human COX IV | 61.9 %  NP_999866.1 | ~ 19.3 kDa  (https://www.uniprot.org/uniprot/Q6TNV0) |
| 6. UQCRC2 | Polyclonal, immunopurified, raised in rabbit | Recombinant fusion protein containing a sequence corresponding to amino acids 224-453 of human UQCRC2 | 61.74 %  NP_001001589.1 | ~ 48 kDa  (https://www.uniprot.org/uniprot/A7YYB3) |
| 7. ATP5A1 | Polyclonal, immunopurified, raised in rabbit | Recombinant fusion protein containing a sequence corresponding to amino acids 1-280 of human ATP5A1 | 88.53 %  NP_001070823.1 | ~ 59.7 kDa  (https://www.uniprot.org/uniprot/Q08BA1) |
| 8. p-IGF-1 Receptor β (Tyr1135/1136) | Monoclonal, immunopurified, raised in rabbit | A synthetic phosphopeptide corresponding to residues surrounding Tyr1135/1136 of human IGF-1 receptor β | 100 %  NP_694501.1 | ~ 152 kDa (precursor)  ~ 95 kDa (beta)  (https://www.uniprot.org/uniprot/A0A2R8Q206) |
| 9. p-IRS1 (Tyr612) | Monoclonal, immunopurified, raised in rabbit | KLH-conjugated linear peptide corresponding to 11 amino acids surrounding phospho tyrosine 612 of human Insulin receptor substrate 1 (IRS1) | 83.33 %  XP_687702.4 | ~ 119 kDa  (https://www.uniprot.org/uniprot/E7F4P8) |
| 10. p-PI3 Kinase p85 (Tyr458) | Polyclonal, immunopurified, raised in rabbit | A synthetic phosphopeptide corresponding to residues surrounding Tyr458 of mouse p85 | 100 %  NP_001268773.1 | ~ 83.7 kDa  (https://www.uniprot.org/uniprot/B0S6V7) |
| 11. p-Akt (Ser473) | Monoclonal, immunopurified, raised in rabbit | A synthetic phosphopeptide corresponding to residues around Ser473 of human Akt | 100 %  NP_001268730.1 | ~ 54.8 kDa  (https://www.uniprot.org/uniprot/M4MD44) |
| 12. p-Akt (Thr308) | Polyclonal, immunopurified, raised in rabbit | A synthetic phosphopeptide (KLH-coupled) corresponding to residues around Thr308 of mouse Akt | 100 %  NP_001268730.1 | ~ 54.8 kDa  (https://www.uniprot.org/uniprot/M4MD44) |
| 13. p-AMPKα (Thr172) | Monoclonal, immunopurified, raised in rabbit | A synthetic phosphopeptide corresponding to residues surrounding Thr172 of human AMPKα protein | 100 %  XP_700831.4 | ~ 62.4 kDa  (https://www.uniprot.org/uniprot/E7F9C4) |
| 14. p-GSK-3β (Ser9) | Monoclonal, immunopurified, raised in rabbit | A synthetic phosphopeptide corresponding to the sequence of human GSK3β | 100 %  NP_571456.1 | ~ 46.8 kDa  (https://www.uniprot.org/uniprot/Q9IBD2) |
| 15. GSK-3β | Monoclonal, immunopurified, raised in rabbit | A synthetic peptide corresponding to the sequence of human GSK3β | 94.76 %  NP_571456.1 | ~ 46.8 kDa  (https://www.uniprot.org/uniprot/Q9IBD2) |
| 16. SIRT1 | Polyclonal, immunopurified, raised in rabbit | Synthetic peptide located within the following region: PETIPPPELDDMPLWQIVINILSEPPKRKKRKDINTIEDAVKLLQECKKI | 79.59 %  XP_001334440.4 | ~ 79.4 kDa  (https://www.uniprot.org/uniprot/E7F8W3) |
| 17. STAR | Polyclonal, immunopurified, raised in rabbit | Recombinant protein of human STAR | 63.6 %  NP_571738.1 | ~ 31.7 kDa  (https://www.uniprot.org/uniprot/Q9DG10) |
| 18. LHCGR | Polyclonal, immunopurified, raised in rabbit | Recombinant fusion protein containing a sequence corresponding to amino acids 200-360 of human LHCGR | 42.24 %  NP_991188.1 | ~ 78 kDa  (https://www.uniprot.org/uniprot/Q7ZVK8) |
| 19. Actin | Polyclonal, immunopurified, raised in rabbit | Synthetic peptide corresponding to C-terminal 11 residues (aa: SGPSIVHRKCF) | 100 %  NP_571106.2 | ~ 41.7 kDa  (<https://www.uniprot.org/uniprot/B2GS08>) |

**Table S2. Oligonucleotide primers and gene accession numbers used in the quantitative RT-PCR assay.**

| Gene | Sequence (5′-3′) | Accession No. |
| --- | --- | --- |
| *PGC-1α* | F: AATGCCAGTGATCAGAGCTGTCCTT  R: GTTCTGTGCCTTGCCACCTGGGTAT | XM017357139.2 ^a^ |
| *PGC-1β* | F: AAGCCAGTATGGGGAAGAGG  R: CCAGCGCTGTACTGTATGGA | XM_009291061.3 ^b^ |
| *NRF-1* | F: AGGCCCTGAGGACTATCGTT  R: GCTCCAGTGCCAACCTGTAT | NM_001328540.1 ^b^ |
| *sdha* | F: TGGTATGCCGTTCAGCCGTA  R: GGCCAAGTCTTTGGCATTGG | NM_200910 ^c^ |
| *uqcrc2* | F: GACCTCACGGGAAGGGTGAA  R: TCAGTGTGCTGGTGCTGCTG | NM_001001589 ^c^ |
| *atp5α1* | F: TTCTTGGAGCCGACACTGGA  R: CGAACACCACAACACCAACG | NM_001077355 ^c^ |
| *TFAM* | F: GCGAAAGATTGCCCAGCAGT  R: TTGTCGTTTTTCCTCCGCAAA | NM_001077389 ^c^ |
| *StAR* | F: ACCTGTTTTCTGGCTGGGATG  R: GGGTCCATTCTCAGCCCTTAC | NM131663 ^d^ |
| *P450scc* | F: AGGGCCATCACCCCAATAG  R: CCAGGCCTTCCCTTCTTTTAG | AF527755 ^d^ |
| *3β-hsd* | F: GCAACTCTGGTTTTCCACACT  R: CAGCAGGAGCCGTGTAGCTT | NM_212797.1 ^e^ |
| *20β-hsd* | F: TGCACGAGTGGTCAATGTGTC  R: ACTAGCTGTCCATGCGGCTCT | NM194379.1 ^d^ |
| *lhcgr* | F: GACGGCCTGAAAGGAGTAAG  R: GCGCAGATTCAGGTTATCAC | NM205625.1 ^d^ |
| *SOD2* | F: TCTCCCTGACCTCACATATGACT  R: TGGCAGCTGATATCTTCTCTTTC | BC060895 ^f^ |
| *HSP70* | F: ACTCTACCAGGGAGGGATGC  R: GTGAACCAACACACTGTGCAATA | AB062116.1 ^f^ |
| *igf1* | F: CAGCAAACCGACAGGATATGG  R: CAGCTCTGAAAGCAGCATTCG | NM131825 ^f^ |
| *igf3* | F: ACGCTGCGGACGAGAACTAG  R: GCTGCTCCAGGTTTGCCTAT | NM131433 ^f^ |
| *ef1α* | F: GTACTACTCTTCTTGATGCCC  R: GTACAGTTCCAATACCTCCA | AY422992 ^f^ |

^a^(Chambers et al., 2018)

^b^(Little et al., 2013)

^c^(Artuso et al., 2012)

^d^(Biswas et al., 2020)

^e^(Ings and Van Der Kraak, 2006)

^f^(Biswas and Maitra, 2021)

**Table S3. Oligonucleotide primers and gene accession numbers used for the quantification of mitochondrial DNA copy number.**

| Gene | Sequence (5′-3′) | Accession No. |
| --- | --- | --- |
| *ndi* | F: TACAGAGGGGGAATCAGAAC  R: TTGGTCGTATCGGAATCGT | AC024175 ^a^ |
| *coxI* | F: GGATTTGGAAACTGACTTGTG  R: AAGAAGAAATGAGGGTGGAAG | AC024175 ^a^ |
| *coxII* | F: TTCCGGCCATCATTCTTATT  R: GTGTGAGGTCTTGGGTTGGT | AC024175 ^b^ |
| *ef1α* | F: GGATTGCCACACTGCCCATA  R: AAGATGACACTTACCCAGAGGA | NC_007124.7 |

^a^(Jin et al., 2010)

^b^(Little et al., 2013)

**REFERENCES**

Artuso, L., Romano, A., Verri, T., Domenichini, A., Argenton, F., Santorelli, F. M., et al. (2012). Mitochondrial DNA metabolism in early development of zebrafish (Danio rerio). *Biochim. Biophys. Acta - Bioenerg.* 1817, 1002–1011. doi: 10.1016/j.bbabio.2012.03.019.

Biswas, S., Ghosh, S., Samanta, A., Das, S., Mukherjee, U., and Maitra, S. (2020). Bisphenol A impairs reproductive fitness in zebrafish ovary: Potential involvement of oxidative/nitrosative stress, inflammatory and apoptotic mediators. *Environ. Pollut.* 267, 115692. doi: 10.1016/j.envpol.2020.115692.

Biswas, S., and Maitra, S. (2021). Altered redox homeostasis in steroid-depleted follicles attenuates hCG regulation of follicular events: Cross-talk between endocrine and IGF axis in maturing oocytes. *Free Radic. Biol. Med.* 172, 675–687. doi: 10.1016/j.freeradbiomed.2021.07.023.

Chambers, J. M., Poureetezadi, S. J., Addiego, A., Lahne, M., and Wingert, R. A. (2018). Ppargc1a controls nephron segmentation during zebrafish embryonic kidney ontogeny. *Elife* 7, 1–22. doi: 10.7554/eLife.40266.

Ings, J. S., and Van Der Kraak, G. J. (2006). Characterization of the mRNA Expression of StAR and Steroidogenic Enzymes in Zebrafish Ovarian Follicles. *Mol. Reprod. Dev. Inc. Gamete Res.* 73, 943–954. doi: https://doi.org/10.1002/mrd.20490.

Jin, Y., Zhang, X., Shu, L., Chen, L., Sun, L., Qian, H., et al. (2010). Oxidative stress response and gene expression with atrazine exposure in adult female zebrafish (Danio rerio). *Chemosphere* 78, 846–852. doi: 10.1016/j.chemosphere.2009.11.044.

Little, A. G., Kunisue, T., Kannan, K., and Seebacher, F. (2013). Thyroid hormone actions are temperature-specific and regulate thermal acclimation in zebrafish (Danio rerio). *BMC Biol.* 11, 1–15. doi: 10.1186/1741-7007-11-26.
